# Thoracic and cardiovascular surgery in Japan during 2012: Annual report by The Japanese Association for Thoracic Surgery

**DOI:** 10.1007/s11748-014-0464-0

**Published:** 2014-10-30

**Authors:** Committee for Scientific Affairs, The Japanese Association for Thoracic Surgery, Munetaka Masuda, Hiroyuki Kuwano, Meinoshin Okumura, Jun Amano, Hirokuni Arai, Shunsuke Endo, Yuichiro Doki, Junjiro Kobayashi, Noboru Motomura, Hiroshi Nishida, Yoshikatsu Saiki, Fumihiro Tanaka, Kazuo Tanemoto, Yasushi Toh, Hiroyasu Yokomise

**Affiliations:** 1 Department of Surgery, Yokohama City University, Yokohama, Japan; 2 Department of General Surgical Science (Surgery I) Gunma University, Graduate School of Medicine, Gunma, Japan; 3 Department of General Thoracic Surgery, Osaka University Graduate School of Medicine, Osaka, Japan; 4 Department of Cardiovascular Surgery, Fujimi-Kogen Medical Center, Nagano, Japan; 5 Department of Cardiovascular Surgery, Tokyo Medical and Dental University Graduate School of Medical and Dental Sciences, Tokyo, Japan; 6 Department of Thoracic Surgery, Jichi Medical University, Tochigi, Japan; 7 Department of Gastroenterological Surgery Graduate School of Medicine, Osaka University, Osaka, Japan; 8 Department of Cardiovascular Surgery, National Cerebral and Cardiovascular Center, Osaka, Japan; 9 Department of Cardiovascular Surgery, Toho University, Sakura Medical Center, Chiba, Japan; 10 Department of Cardiovascular Surgery, The Heart Institute of Japan, Tokyo Women’s Medical University, Tokyo, Japan; 11 Division of Cardiovascular Surgery, Tohoku University Graduate School of Medicine, Miyagi, Japan; 12 Second Department of Surgery, University of Occupational and Environmental Health, Japan, Fukuoka, Japan; 13 Department of Cardiovascular Surgery, Kawasaki Medical School, Okayama, Japan; 14 Department of Gastroenterological Surgery, National Kyushu Cancer Center, Fukuoka, Japan; 15 Department of General Thoracic Surgery, Faculty of Medicine, Kagawa University, Kagawa, Japan; 16 Tokyo, Japan

The Japanese Association for Thoracic Surgery has conducted annual surveys of thoracic surgery throughout Japan since 1987 to determine the statistics regarding the number of procedures according to operative category. Here, we have summarized the results from our annual survey of thoracic surgery performed during 2012.

**Table 1:** **Table 1** Questionnaires sent out and received back by the end of December 2013

	Sent out	Returned	Response rate (%)
(A) Cardiovascular surgery	601	583	97.0
(B) General thoracic surgery	802	777	96.9
(C) Esophageal surgery	582	555	95.4

**Table 2:** **Table 2** Categories subclassified according to the number of operations performed

Number of operations performed	Category	
	Cardiovascular surgery	General thoracic surgery
0	39	41
1–24	46	92
25–49	99	92
50–99	163	193
100–149	86	134
150–199	60	107
≧200	90	118
Total	583	777

Number of operations performed	Esophageal surgery
0	86
1–4	99
5–9	81
10–19	105
20–29	48
30–39	35
40–49	30
≧50	71
Total	555

**Fig. 1:**
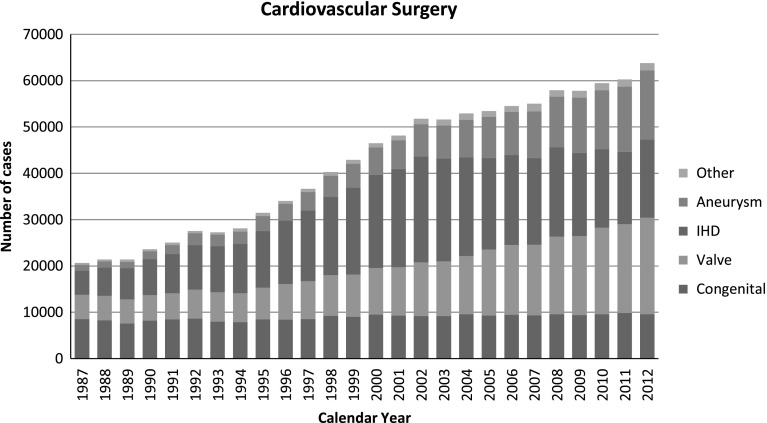
Cardiovascular surgery, *IHD* ischemic heart disease

The incidence of hospital mortality was added to the survey to determine the nationwide status, which has contributed to the Japanese surgeons to understand the present status of thoracic surgery in Japan and to make progress to improve operative results by comparing their work with those of others. The Association was able to gain a better understanding of the present problems as well as future prospects, which has been reflected to its activity including education of its members. Thirty-day mortality (so-called “operative mortality) is defined as death within 30 days of operation regardless of the patient’s geographic location and even though the patient had been discharged from the hospital.

Hospital mortality is defined as death within any time interval after an operation if the patient had not been discharged from the hospital. Hospital-to-hospital transfer is not considered discharge: transfer to a nursing home or a rehabilitation unit is considered hospital discharge unless the patient subsequently dies of complications of the operation. The definitions of the Ad Hoc Liaison Committee for Standardizing Definitions of Prosthetic Heart Valve Morbidity of the Society of Thoracic Surgeons and the American Association for Thoracic Surgery (Edmunds et al. Ann Thorac Surg 1996;62:932–5; J Thorac Cardiovasc Surg 1996;112:708–11).

Thoracic surgery was classified into three categories—cardiovascular, general thoracic, and esophageal surgery—and the patient data were examined and analyzed for each group. Access to the computerized data is offered to all members of this Association. We honor and value all member’s continued kind support and contributions (Tables 1, 2).

**Table:** **Table 1** Congenital (total; 9,558) (1) CPB (+) (total; 7,171)

		Neonate				Infant				1–17 years				≧18 years				Total			
		Cases	30-day mortality		Hospital mortality	Cases	30-day mortality		Hospital mortality	Cases	30-day mortality		Hospital mortality	Cases	30-day mortality		Hospital mortality	Cases	30-day mortality		Hospital mortality
			Hospital	After discharge			Hospital	After discharge			Hospital	After discharge			Hospital	After discharge			Hospital	After discharge	
1	PDA	15	0	0	0	2	0	0	0	4	0	0	0	24	1 (4.2)	0	1 (4.2)	45	1 (2.2)	0	1 (2.2)
2	Coarctation (simple)	7	0	0	0	13	0	0	0	17	0	0	0	7	0	0	0	44	0	0	0
3	+VSD	32	2 (6.3)	0	2 (6.3)	34	0	0	0	8	0	0	0	2	0	0	0	76	2 (2.6)	0	2 (2.6)
4	+DORV	2	0	0	1 (50.0)	3	0	0	0	2	0	0	0	0	0	0	0	7	0	0	1 (14.3)
5	+AVSD	5	0	0	0	4	0	0	0	1	0	0	0	0	0	0	0	10	0	0	0
6	+TGA	3	1 (33.3)	0	1 (33.3)	3	0	0	0	1	0	0	0	0	0	0	0	7	1 (14.3)	0	1 (14.3)
7	+SV	9	2 (22.2)	0	2 (22.2)	7	0	0	0	4	0	0	0	0	0	0	0	20	2 (10.0)	0	2 (10.0)
8	+Others	2	0	0	0	5	0	0	0	4	0	0	0	1	0	0	0	12	0	0	0
9	Interrupt. of Ao (simple)	1	0	0	0	2	1 (50.0)	0	1 (50.0)	2	0	0	0	1	0	0	0	6	1 (16.7)	0	1 (16.7)
10	+VSD	25	2 (8.0)	0	2 (8.0)	16	0	0	0	4	0	0	0	10	0	0	0	55	2 (3.6)	0	2 (3.6)
11	+DORV	3	1 (33)	0	1 (33.3)	4	0	0	0	2	0	0	0	0	0	0	0	9	1 (11.1)	0	1 (11.1)
12	+Truncus	2	1 (50.0)	0	1 (50.0)	2	0	0	0	0	0	0	0	0	0	0	0	4	1 (25.0)	0	1 (25.0)
13	+TGA	2	0	0	0	1	0	0	0	0	0	0	0	0	0	0	0	3	0	0	0
14	+Others	4	0	0	0	10	1 (10.0)	0	1 (10.0)	2	0	0	0	1	0	0	0	17	1 (5.9)	0	1 (5.9)
15	Vascular ring	0	0	0	0	4	0	0	0	3	0	0	0	0	0	0	0	7	0	0	0
16	PS	1	0	0	0	15	0	0	0	14	0	0	0	4	0	0	1 (25.0)	34	0	0	1 (2.9)
17	PAIVS or critical PS	12	0	0	0	50	1 (2.0)	0	1 (2.0)	55	0	0	0	2	0	0	1 (50.0)	119	1 (0.8)	0	2 (1.7)
18	TAPVR	126	9 (7.1)	0	15 (11.9)	49	1 (2)	2 (4.08)	2 (4.1)	8	0	0	0	0	0	0	0	183	10 (5)	2 (1.1)	17 (9)
19	PAPVR ± ASD	0	0	0	0	4	0	0	0	52	0	0	0	29	0	0	0	85	0	0	0
20	ASD	11	1 (9.1)	0	1 (9.1)	54	0	0	0	693	0	0	0	573	2 (0.3)	1 (0.2)	2 (0.3)	1,331	3 (0.2)	1 (0.08)	3 (0.2)
21	Cor triatriatum	1	0	0	0	13	2 (15.4)	0	2 (15.4)	3	0	0	0	4	0	0	0	21	2 (9.5)	0	2 (9.5)
22	AVSD (partial)	1	1 (100.0)	0	1 (100.0)	19	1 (5.3)	0	1 (5.3)	40	0	0	0	13	0	0	0	73	2 (2.7)	0	2 (2.7)
23	AVSD (complete)	4	0	0	1 (25.0)	108	5 (4.6)	0	5 (4.6)	69	0	0	0	4	0	0	0	185	5 (2.7)	0	6 (3.2)
24	+TOF or DORV	0	0	0	0	6	1 (16.7)	0	2 (33.3)	21	0	0	0	3	0	0	0	30	1 (3.3)	0	2 (6.7)
25	+Others	1	0	0	0	10	1 (10.0)	0	1 (10.0)	8	0	0	0	1	0	0	0	20	1 (5.0)	0	1 (5.0)
26	VSD (subarterial)	3	0	0	0	128	0	0	0	197	0	0	0	29	0	0	0	357	0	0	0
27	VSD (perimemb./muscular)	10	0	0	0	770	0	1 (0.13)	0	390	0	0	0	90	0	0	0	1,260	0	1 (0.1)	0
28	VSD + PS	0	0	0	0	39	0	0	0	33	0	0	0	7	0	0	0	79	0	0	0
29	DCRV ± VSD	2	0	0	0	16	0	0	0	39	0	0	0	19	0	0	0	76	0	0	0
30	Aneurysm of sinus valsalva	0	0	0	0	6	0	0	0	1	0	0	0	24	0	0	0	31	0	0	0
31	TOF	15	1 (6.7)	0	1 (6.7)	168	0	0	0	231	2 (0.9)	0	2 (0.9)	22	0	0	2 (9.1)	436	3 (0.7)	0	5 (1.1)
32	PA + VSD	4	0	0	0	78	1 (1.3)	0	1 (1.3)	125	3 (2.4)	0	5 (4.0)	9	0	0	0	216	4 (1.9)	0	6 (2.8)
33	DORV	17	1 (5.9)	0	1 (5.9)	91	1 (1.1)	0	3 (3.3)	112	2 (1.8)	0	2 (1.8)	3	1 (33.3)	0	1 (33.3)	223	5 (2.2)	0	7 (3.1)
34	TGA (simple)	101	2 (2.0)	0	3 (3.0)	12	0	0	0	0	0	0	0	4	0	0	0	117	2 (1.7)	0	3 (2.6)
35	+VSD	44	1 (2.3)	0	1 (2.3)	17	1 (5.9)	0	1 (5.9)	2	0	0	0	0	0	0	0	63	2 (3.2)	0	2 (3.2)
36	VSD + PS	4	0	0	0	8	0	0	0	26	0	0	0	2	0	0	0	40	0	0	0
37	Corrected TGA	2	0	0	0	11	0	0	0	42	1 (2.4)	0	1 (2.4)	13	0	0	0	68	1 (1.5)	0	1 (1.5)
38	Truncus arteriosus	4	0	0	0	26	2 (7.7)	0	3 (11.5)	12	0	0	0	1	0	0	0	43	2 (4.7)	0	3 (7.0)
39	SV	27	5 (18.5)	0	8 (29.6)	219	7 (3.2)	0	11 (5.0)	282	4 (1.4)	0	9 (3.2)	21	2 (9.5)	0	2 (9.5)	549	18 (3.3)	0	30 (5.5)
40	TA	2	0	0	0	30	0	0	0	53	0	0	0	5	0	0	0	90	0	0	0
41	HLHS	45	10 (22.2)	0	10 (22.2)	130	11 (8.5)	1 (0.77)	15 (11.5)	71	0	0	0	0	0	0	0	246	21 (8.5)	1 (11.7)	25 (10.2)
42	Aortic valve lesion	7	3 (42.9)	0	3 (42.9)	16	0	0	0	75	3 (4.0)	0	3 (4.0)	19	1 (5.3)	0	1 (5.3)	117	7 (6.0)	0	7 (6.0)
43	Mitral valve lesion	2	1 (50.0)	0	1 (50.0)	56	0	0	2 (3.6)	75	1 (1.3)	0	1 (1.3)	14	0	0	0	147	2 (1.4)	0	4 (2.7)
44	Ebstein	10	1 (10.0)	0	1 (10.0)	17	1 (5.9)	0	1 (5.9)	31	0	0	0	12	0	0	0	70	2 (2.9)	0	2 (2.9)
45	Coronary disease	0	0	0	0	11	0	0	0	16	0	0	0	13	0	0	0	40	0	0	0
46	Others	11	0	0	1 (9.1)	27	3 (11.1)	0	3 (11.1)	34	2 (5.9)	0	2 (5.9)	14	0	0	0	86	5 (5.8)	0	6 (7.0)
47	Redo VSD	0	0	0	0	6	0	0	0	10	0	0	0	7	0	0	0	23	0	0	0
48	PS release	0	0	0	0	8	0	0	0	42	0	0	0	23	0	0	0	73	0	0	0
49	RV-PA conduit replace	0	0	0	0	3	0	0	0	68	1 (1.5)	0	1(1.5)	17	0	0	1 (5.9)	88	1 (1.1)	0	2 (2.3)
50	Others	1	0	0	0	62	6 (9.7)	0	7 (11.3)	121	2 (1.7)	0	4 (3.3)	46	1 (2.2)	0	1 (2.2)	230	9 (3.9)	0	12 (5.2)
Total		580	45 (7.8)	0	58 (10.0)	2,393	47 (2.0)	4 (0.17)	63 (2.6)	3,105	21 (0.7)	0	30 (1.0)	1,093	8 (0.7)	1 (0.1)	13 (1.2)	7,171	121 (1.7)	5 (0.1)	164 (2.3)

Values in parenthesis represent mortality %

*CPB* cardiopulmonary bypass, *PDA* patient ductus arteriosus, *VSD* ventricular septal defect, *DORV* double outlet right ventricle, *AVSD* atrioventricular septal defect, *TGA* transposition of great arteries, *SV* single ventricle, *Interupt. of Ao.*, interrupted aortic arch, *PS* pulmonary stenosis, *PA-IVS* pulmonary atresia with intact ventricular septum, *TAPVR* total anomalous pulmonary venous return, *PAPVR* partial anomalous pulmonary venous return, *ASD* atrial septal defect, *TOF* tetralogy of Fallot, *DCRV* double-chambered right ventricle, *TA* tricuspid atresia, *HLHS* hypoplastic left heart syndrome, *RV-PA* right ventricle–pulmonary artery

**Table:** (2) CPB (−) (total; 2,387)

		Neonate				Infant				1–17 years				≧18 years				Total			
		Cases	30-day mortality		Hospital mortality	Cases	30-day mortality		Hospital mortality	Cases	30-day mortality		Hospital mortality	Cases	30-day mortality		Hospital mortality	Cases	30-day mortality		Hospital mortality
			Hospital	After discharge			Hospital	After discharge			Hospital	After discharge			Hospital	After discharge			Hospital	After discharge	
1	PDA	358	2 (0.6)	0	3 (0.8)	225	0	0	2 (0.9)	46	0	0	0	1	0	0	0	630	2 (0.3)	0	5 (0.8)
2	Coarctation (simple)	21	0	0	0	14	0	0	0	4	0	0	0	1	0	0	0	40	0	0	0
3	+VSD	38	1 (2.6)	1 (2.6)	1 (2.6)	26	1 (3.8)	0	1 (3.8)	0	0	0	0	0	0	0	0	64	2 (3.1)	1 (1.6)	2 (3.1)
4	+DORV	5	0	0	1 (20.0)	2	0	0	0	0	0	0	0	0	0	0	0	7	0	0	1 (14.3)
5	+AVSD	5	1 (20.0)	0	1 (20.0)	0	0	0	0	0	0	0	0	0	0	0	0	5	1 (20.0)	0	1
6	+TGA	7	0	0	0	0	0	0	0	0	0	0	0	0	0	0	0	7	0	0	0
7	+SV	5	0	0	0	2	0	0	0	0	0	0	0	0	0	0	0	7	0	0	0
8	+Others	4	0	0	0	2	0	0	0	0	0	0	0	0	0	0	0	6	0	0	0
9	Interrupt. of Ao (simple)	1	0	0	0	0	0	0	0	0	0	0	0	0	0	0	0	1	0	0	0
10	+VSD	21	0	0	1 (4.8)	5	0	0	0	2	0	0	0	0	0	0	0	28	0	0	1 (3.6)
11	+DORV	4	1 (25)	0	2 (50)	0	0	0	0	0	0	0	0	0	0	0	0	4	1 (25)	0	2 (50)
12	+Truncus	3	0	0	0	0	0	0	0	0	0	0	0	0	0	0	0	3	0	0	0
13	+TGA	2	0	0	0	1	0	0	0	0	0	0	0	0	0	0	0	3	0	0	0
14	+Others	7	0	0	1 (14.3)	0	0	0	0	0	0	0	0	0	0	0	0	7	0	0	1 (14.3)
15	Vascular ring	1	0	0	0	12	1 (8.3)	0	1 (8.3)	9	0	0	0	0	0	0	0	22	1 (4.5)	0	1 (4.5)
16	PS	0	0	0	0	1	0	0	0	0	0	0	0	0	0	0	0	1	0	0	0
17	PAIVS or critical PS	30	3 (10.0)	0	3 (10.0)	26	0	0	0	3	0	0	0	2	0	0	0	61	3 (4.9)	0	3 (4.9)
18	TAPVR	1	0	0	0	2	0	0	0	0	0	0	0	0	0	0	0	3	0	0	0
19	PAPVR ± ASD	0	0	0	0	0	0	0	0	0	0	0	0	1	0	0	0	1	0	0	0
20	ASD	0	0	0	0	0	0	0	0	12	0	0	0	15	0	0	0	27	0	0	0
21	Cor triatriatum	0	0	0	0	0	0	0	0	0	0	0	0	0	0	0	0	0	0	0	0
22	AVSD (partial)	2	0	0	0	4	0	0	0	1	0	0	0	0	0	0	0	7	0	0	0
23	AVSD (complete)	32	0	0	0	68	0	0	1 (1.5)	4	0	0	0	0	0	0	0	104	0	0	1 (1.0)
24	+TOF or DORV	3	0	0	0	13	1 (7.7)	0	1 (7.7)	6	0	0	0	0	0	0	0	22	1 (4.5)	0	1 (4.5)
25	+Others	3	1 (33.3)	0	1 (33.3)	4	0	0	0	2	0	0	0	0	0	0	0	9	1 (11.1)	0	1 (11.1)
26	VSD (subarterial)	2	0	0	0	11	0	0	0	2	0	0	0	2	0	0	0	17	0	0	0
27	VSD (perimemb./muscular)	38	0	0	0	117	1 (0.9)	0	3 (2.6)	5	0	0	0	1	0	0	0	161	1 (0.6)	0	3 (1.9)
28	VSD + PS	0	0	0	0	1	0	0	0	0	0	0	0	0	0	0	0	1	0	0	0
29	DCRV ± VSD	0	0	0	0	0	0	0	0	0	0	0	0	0	0	0	0	0	0	0	0
30	Aneurysm of sinus valsalva	1	0	0	0	0	0	0	0	0	0	0	0	1	0	0	0	2	0	0	0
31	TOF	24	0	0	0	100	2 (2.0)	0	2 (2.0)	13	0	0	0	4	0	0	0	141	2 (1.4)	0	2 (1.4)
32	PA + VSD	26	0	0	0	83	0	0	0	15	0	0	0	1	0	0	0	125	0	0	0
33	DORV	27	0	0	0	55	1 (1.8)	0	2 (3.6)	14	0	0	0	2	0	0	0	98	1 (1.0)	0	2 (2.0)
34	TGA (simple)	7	0	0	0	1	0	0	0	1	0	0	0	1	0	0	0	10	0	0	0
35	+VSD	7	0	0	0	3	0	0	0	0	0	0	0	0	0	0	0	10	0	0	0
36	VSD + PS	9	1 (11.1)	0	1 (11.1)	5	0	0	0	2	0	0	0	0	0	0	0	16	1 (6.3)	0	1 (6.3)
37	Corrected TGA	8	0	0	0	18	1 (5.6)	0	1 (5.6)	4	0	0	0	0	0	0	0	30	1 (3.3)	0	1 (3.3)
38	Truncus arteriosus	20	0	0	1 (5.0)	1	0	0	0	5	0	0	0	0	0	0	0	26	0	0	1 (3.8)
39	SV	72	2 (2.8)	0	3 (4.2)	57	1 (1.8)	0	3 (5.3)	21	1 (4.8)	0	1	3	1 (33.3)	0	1 (33.3)	153	5 (3.3)	0	8 (5.2)
40	TA	18	0	0	0	20	0	0	0	12	0	0	0	1	0	0	0	51	0	0	0
41	HLHS	81	2 (2.5)	0	3 (3.7)	19	0	0	0	3	0	0	0	0	0	0	0	103	2 (1.9)	0	3 (2.9)
42	Aortic valve lesion	4	0	0	0	2	0	0	0	4	0	0	0	2	0	0	0	12	0	0	0
43	Mitral valve lesion	0	0	0	0	0	0	0	0	1	0	0	0	0	0	0	0	1	0	0	0
44	Ebstein	6	1 (16.7)	0	2 (33.3)	4	0	0	0	1	0	0	0	3	0	0	0	14	1 (7.1)	0	2 (14.3)
45	Coronary disease	1	1 (100.0)	0	1 (100.0)	0	0	0	0	2	0	0	0	1	0	0	0	4	1 (25.0)	0	1 (25.0)
46	Others	24	1 (4.2)	0	1 (4.2)	65	1 (1.5)	0	1 (1.5)	75	1 (1.3)	0	1	23	0	0	0	187	3 (1.6)	0	3 (1.6)
47	Redo VSD	0	0	0	0	3	0	0	0	36	0	0	0	1	0	0	0	40	0	0	0
48	PS release	0	0	0	0	0	0	0	0	1	0	0	0	0	0	0	0	1	0	0	0
49	RV-PA conduit replace	0	0	0	0	0	0	0	0	0	0	0	0	0	0	0	0	0	0	0	0
50	Others	18	0	0	1 (5.6)	36	0	0	0	45	0	0	0	16	1 (6.3)	0	1 (6.3)	115	1 (0.9)	0	2 (1.7)
Total		946	17 (1.8)	1 (0.1)	27 (2.9)	1,008	10 (1.0)	0	18 (1.8)	351	2 (0.6)	0	2	82	2 (2.4)	0	2 (2.4)	2,387	31 (1.3)	1 (0.04)	49 (2.1)

Values in parenthesis represent mortality %

*CPB* cardiopulmonary bypass, *PDA* patient ductus arteriosus, *VSD* ventricular septal defect, *DORV* double outlet right ventricle, *AVSD* atrioventricular septal defect, *TGA* transposition of great arteries, *SV* single ventricle, *Interupt. of Ao.* interrupted aortic arch, *PS* pulmonary stenosis, *PA-IVS* pulmonary atresia with intact ventricular septum, *TAPVR* total anomalous pulmonary venous return, *PAPVR* partial anomalous pulmonary venous return, *ASD* atrial septal defect, *TOF* tetralogy of Fallot, *DCRV* double-chambered right ventricle, *TA* tricuspid atresia, *HLHS* hypoplastic left heart syndrome, *RV-PA* right ventricle–pulmonary artery

**Table:** (3) Main procedure

		Neonate				Infant				1–17 years				≧18 years				Total			
		Cases	30-day mortality		Hospital mortality	Cases	30-day mortality		Hospital mortality	Cases	30-day mortality		Hospital mortality	Cases	30-day mortality		Hospital mortality	Cases	30-day mortality		Hospital mortality
			Hospital	After discharge			Hospital	After discharge			Hospital	After discharge			Hospital	After discharge			Hospital	After discharge	
1	SP shunt	170	5 (2.9)	0	9 (5.3)	425	9 (2.1)	0	12 (2.8)	58	1 (1.7)	0	1 (1.7)	1	0	0	0	654	15 (2.3)	0	22 (3.4)
2	PAB	359	6 (1.7)	0	10 (2.8)	250	3 (1.2)	0	5 (2.0)	15	0	0	0	2	0	0	0	626	9 (1.4)	0	15 (2.4)
3	Bidirectional Glenn or hemi-Fontan ± α	0	0	0	0	265	6 (2.3)	0	6 (2.3)	106	0	0	2 (1.9)	4	0	0	0	375	6 (1.6)	0	8 (2.1)
4	Damus–Kaye–Stansel operation	4	1 (25.0)	0	2	45	2	0	2	21	0	0	0	0	0	0	0	70	3 (4.3)	0	4 (5.7)
5	PA reconstruction/repair (including redo)	9	0	0	0	99	1 (1.0)	0	2 (2.0)	108	0	0	1 (0.9)	18	0	0	1 (5.6)	234	1 (0.4)	0	4 (1.7)
6	RVOT reconstruction/repair	16	2 (12.5)	0	2 (12.5)	107	0	0	1 (0.9)	231	2 (0.9)	0	2 (0.9)	17	0	0	0	371	4 (1.1)	0	5 (1.3)
7	Rastelli procedure	3	1 (33.3)	0	1 (33.3)	44	2 (4.5)	0	2 (4.5)	108	2 (1.9)	0	3 (2.8)	10	0	0	0	165	5 (3.0)	0	6 (3.6)
8	Arterial switch procedure	154	5 (3.2)	0	8 (5.2)	23	1 (4.3)	0	1 (4.3)	6	0	0	0	0	0	0	0	183	6 (3.3)	0	9 (4.9)
9	Atrial switch procedure	4	0	0	0	0	0	0	0	2	0	0	0	1	0	0	0	7	0	0	0
10	Double switch procedure	0	0	0	0	0	0	0	0	11	0	0	0	0	0	0	0	11	0	0	0
11	Repair of anomalous origin of CA	1	0	0	0	6	0	0	0	14	0	0	0	7	0	0	0	28	0	0	0
12	Closure of coronary AV fistula	0	0	0	0	1	0	0	0	5	0	0	1 (20.0)	24	0	0	0	30	0	0	1 (3.3)
13	Fontan/TCPC	1	0	0	0	3	0	0	0	408	4 (1.0)	0	6 (1.5)	26	2 (7.7)	0	3 (11.5)	438	6 (1.4)	0	9 (2.1)
14	Norwood procedure	42	8 (19.0)	1 (2.4)	7 (16.7)	78	7 (9.0)	0	11 (14.1)	10	1 (10.0)	0	2 (20.0)	0	0	0	0	130	16 (12.3)	1 (8.1)	20 (15.4)
15	Ventricular septation	0	0	0	0	7	2 (28.6)	0	2 (28.6)	4	0	0	0	1	0	0	0	12	2 (16.7)	0	2 (16.7)
16	Left side AV valve repair (including redo)	0	0	0	0	66	1 (1.5)	0	1 (1.5)	63	1 (1.6)	0	1 (1.6)	11	1 (9.1)	0	1 (9.1)	140	3 (2.1)	0	3 (2.1)
17	Left side AV valve replace (including redo)	1	1 (100)	0	1 (100)	15	1 (6.7)	0	1 (6.7)	41	2 (4.9)	0	2 (4.9)	20	0	0	0	77	4 (5.2)	0	4 (5.2)
18	Right side AV valve repair (including redo)	2	0	0	0	13	0	0	0	34	0	0	0	30	0	0	0	79	0	0	0
19	Right side AV valve replace (including redo)	0	0	0	0	1	0	0	0	6	0	0	0	8	0	0	0	15	0	0	0
20	Common AV valve repair (including redo)	2	1 (50.0)	0	1 (50.0)	34	5 (14.7)	0	5 (14.7)	19	1 (5.3)	0	1 (5.3)	1	0	0	0	56	7 (12.5)	0	7 (12.5)
21	Common AV valve replace (including redo)	2	1 (50.0)	0	1 (50.0)	6	1 (16.7)	0	1 (16.7)	8	0	0	1 (12.5)	3	0	0	0	19	2 (10.5)	0	3 (15.8)
22	Repair of supra-aortic stenosis	1	0	0	0	6	1 (16.7)	0	1 (16.7)	9	1 (11.1)	0	1 (11.1)	0	0	0	0	16	2 (12.5)	0	2 (12.5)
23	Repair of subaortic stenosis (including redo)	1	1 (100.0)	0	1 (100.0)	7	0	0	1 (14)	36	0	0	0	4	0	0	0	48	1 (2.1)	0	2 (4.2)
24	Aortic valve plasty ± VSD closure	3	0	0	0	12	0	0	1 (8.3)	24	0	0	0	4	0	0	0	43	0	0	1 (2.3)
25	Aortic valve replacement	0	0	0	0	2	0	0	0	22	0	0	0	23	0	0	0	47	0	0	0
26	AVR with annular enlargement	1	0	0	0	3	0	0	0	13	0	0	0	6	1 (16.7)	0	1 (16.7)	23	1 (4.3)	0	1 (4.3)
27	Aortic root replace (except Ross)	0	0	0	0	0	0	0	0	6	0	0	0	5	0	0	0	11	0	0	0
28	Ross procedure	0	0	0	0	3	0	0	0	10	0	0	0	1	0	0	0	14	0	0	0
Total		776	32 (4.1)	1 (0.1)	43 (5.5)	1,521	42 (2.8)	0	55 (3.6)	1,398	15 (1.1)	0	24 (1.7)	227	4 (1.8)	0	6 (2.6)	3,922	93 (2.4)	1 (0.03)	128 (3.3)

Values in parenthesis represent mortality %

*SP* systemic pulmonary, *PAB* pulmonary artery banding, *PA* pulmonary artery, *RVOT* right ventricular outflow tract, *CA* coronary artery, *AV* *fustula* arteriovenous fistula, *TCPC* total cavopulmonary connection, *AV valve* atrioventricular valve, *VSD* ventricular septal defect, *AVR* aortic valve replacement

## Abstract of the survey

We sent out survey questionnaire forms to the departments of each category in all 1,986 institutions (601 cardiovascular, 802 general thoracic and 582 esophageal) nationwide in early April 2013. The response rates in each category by the end of December 2013 were 97.0, 96.8, and 95.2 %, respectively. This high response rate has been keep throughout recent survey, and more than 95 % response rate in all fields in 2012 survey has to be congratulated.

## 2012 Final report

### (A) Cardiovascular surgery

First, we are very pleased with the high response rate to our survey of cardiovascular surgery (97.0 %), which definitely enhances the quality of this annual report. We very much appreciate the enormous effort put into completing the survey at each participating institution.

Figure 1 shows the development of cardiovascular surgery in Japan over the last 26 years. Aneurysm surgery includes only operations for thoracic and thoracoabdominal aortic aneurysm. Pacemaker implantation includes only transthoracic implantation and transvenous implantation is excluded. The number of pacemaker and assist device implantation operations is not included in the total number of surgical operations. A total of 63,800 cardiovascular operations were performed at 583 institutions during 2012 alone and included 28 heart transplantations, which were restarted in 1999.

**Fig. 1:**
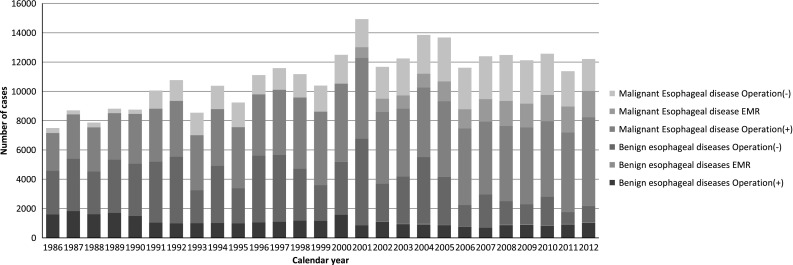
Annual trend of in-patients with esophageal diseases, *EMR* endoscopic mucosal resection (including endoscopic submucosal)

The number of operations for congenital heart disease (9,558 cases) decreased slightly (3.1 %) compared with that of 2011 (9,859 cases), while there was 3.9 % increase when compared with the data of 10 years ago (9,202 cases in 2002). The number of operations for adult cardiac disease (20,913 cases in valvular heart disease, 16,752 cases in ischemic heart disease, 14,944 cases in thoracic aortic aneurysm and 1,663 cases for other procedures) increased compared with those of 2011 in all categories (9.1, 7.5, 5.8 and 5.1 %, respectively). During the last 10 years, the numbers of operations for adult heart disease increased constantly except for that of ischemic heart disease (81.0 % increase in valvular heart disease, 26.6 % decrease in ischemic heart disease, 112.4 % increase in thoracic aortic aneurysm, and 40.7 % increase in other procedures compared those of 2002). The concomitant coronary artery bypass grafting procedure (CABG) is not included in ischemic heart disease but included in other categories such as valvular heart disease in our study, then, the number of CABG still remained over 20,000 cases per year (21,569 cases) in 2012, which is 89.4 % of that in 2002 (24,135 cases).

Data for individual categories are summarized in tables through 1 to 7.

In 2012, 7,171 open-heart operations for congenital heart disease were performed with overall hospital mortality of 2.3 %. The number of operations for congenital heart disease was quite steady throughout these 10 years (maximum 7,386 cases in 2006), while overall hospital mortality decreased gradually from that of 3.6 % in 2002. In detail, the most common disease was atrial septal defect (1,331 cases), however, its number deceased to 71.7 % of that in 2002, which might be due to the recent development of catheter closure of atrial septal defect in Japan. Hospital mortality for complex congenital heart disease improved dramatically in the last 10 years such as interrupted aortic arch with ventricular septal defect (13.9 % in 2002 to 3.6 % in 2012), complete atrio-septal defect (4.2 to 3.2 %), Tetralogy of Fallot (3.8 to 1.1 %), transposition of the great arteries with and without ventricular septal defect (14.0 to 3.2 % and 7.4 to 2.6 %, respectively), single ventricle and tricuspid atresia (9.2 to 5.5 % and 3.9 to 0 %, respectively), and hypoplastic left heart syndrome (37.9 to 10.2 %). Right heart bypass surgery is now commonly performed (375 bidirectional Glenn procedures and 438 Fontan type procedures including total cavopulmonary connection) with acceptable hospital mortality (2.1 % in each procedure). Norwood type I procedure was performed in 130 cases with relatively low hospital mortality rate of 15.4 %.

As previously mentioned, the number of operations for valvular heart disease increased by 81 % in the last 10 years, and the hospital mortality associated with primary single valve replacement was 3.0 and 4.5 % for the aortic and the mitral position, while that for primary mitral valve repair was 1.3 %. However, hospital mortality rate for redo valve surgery was still high and was 9.3 and 6.7 % for aortic and mitral procedure, respectively. Finally, overall hospital mortality did not show any improvement during the last 10 years (3.1 % in 2002 and 3.2 % in 2012), which might be partially due to the recent progression of age of the patients. Repair of the valve became popular procedure (484 cases in the aortic, 6,002 cases in the mitral, and 4,947 case in the tricuspid), and mitral valve repair constituted 28.7 % of all valvular heart disease operation and 57.6 % of all mitral valve procedure (10,425 procedures), which are similar to those of the last 4 years and increased compared with those of 2002 (19.5 and 34.9 %, respectively). Aortic and mitral valve replacement with bioprosthesis were performed in 8,926 cases and 3,002 cases, respectively, with the number consistently increasing. The ratio of prostheses changed dramatically during the last 10 years, and the usage of bioprosthesis is 74.3 % at the aortic position (37.3 % in 2002) and 61.0 % at the mitral position (24.2 % in 2002). CABG as a concomitant procedure increased gradually to 23.9 % of operations for all valvular heart disease (12.1 % in 2002).

Isolated CABG was performed in 15,462 cases which were only 71.5 % of that of 10 years ago (2002), however, there was an increase of 8.5 % compared with that in 2011. Among these 15,462 cases, off-pump CABG was intended in 9,499 cases (61.4 %) with a success rate of 97.9 %, so final success rate of off-pump CABG was 60.2 %. The percentage of intended off-pump CABG was 55.2 % in 2003, and was increased to 60.3 % in 2004, then was kept over 60 % until now. Conversion rate from off-pump CABG to on-pump CABG of 2.1 % was just same as that in 2011. In 15,462 isolated CABG patients, 96.5 % of them received at least one arterial graft, while, all arterial graft CABG was performed in only 23.5 % of them.

The operative and hospital mortality rates associated with primary elective CABG procedures in 13,004 cases were 0.6 and 1.1 %, respectively. Similar data analysis of CABG including primary/redo and elective/emergency data was begun in 2003, and the operative and hospital mortality rates associated with primary elective CABG procedures in 2003 were 1.0 and 1.5 %, respectively, so operative results of primary CABG have been improved. However, hospital mortality of primary emergency CABG in 2,224 cases was 7.4 %, which was still high in spite of slight improvement compared with 9.7 % of hospital mortality rate in 2003. In comparison with data in 2003, the results of conversion improved both conversion rate (3.1 to 2.1 %) and hospital mortality (8.5 to 5.1 %).

A total of 1,274 patients underwent surgery for complications of myocardial infarction, including 413 operations for a left ventricular aneurysm or ventricular septal perforation or cardiac rupture and 296 operations for ischemic mitral regurgitation.

Operations for arrhythmia were performed mainly as a concomitant procedure in 3,992 cases with satisfactory mortality (1.8 % hospital mortality) including 3,771 MAZE procedures. MAZE procedure has become quite popular procedure when compared with that in 2002 (1,141 cases).

Operations for thoracic aortic dissection were performed in 6,266 cases. For 4,186 Stanford type A acute aortic dissections, hospital mortality was 10.6 %, which was similar to that in 2011 (11.1 %) and better than that in 2002 (15.5 %). Operations for a non-dissected thoracic aneurysm were carried out in 8,678 cases, with overall hospital mortality of 5.4 %, which was better than that in 2011 (6.7 %). The hospital mortality associated with unruptured aneurysm was 4.0 %, and that of ruptured aneurysm was 22.2 %, which remains markedly high.

The number of stent graft procedures remarkably increased recently. A total of 835 patients with aortic dissection underwent stent graft placement: thoracic endovascular aortic repair (TEVAR) in 723 cases, open stent grafting in 109 cases, and unspecified in 3 cases. The number of TEVAR for type B chronic aortic dissections increased from 359 cases in 2011 to 492 cases in 2012. The hospital mortality rates associated with TEVAR for type B aortic dissection were 7.3 % in acute cases and 2.6 % for chronic cases, respectively.

A total of 3,236 patients with non-dissected aortic aneurysm underwent stent graft placement with 18.8 % increase compared with that in 2011 (2,725 cases); TEVAR in 3,006 cases (23.6 % increase compared with that in 2011), open stent grafting in 226 cases (20.8 % decrease compared with that in 2011), and unspecified in 4 cases. The hospital mortality rates for TEVAR were 2.5 and 16.1 % for non-ruptured and ruptured aneurysm, respectively.

In summary, the total cardiovascular operations increased during 2012 by 3,516 cases, with steadily improving results in almost all categories compared with those in 2011.

**Table:** **Table 2** Acquired (total, (1) + (2) + (4) + (5) + (6) + (7) + isolated ope. for arrhythmia in (3); 39,177 (1) Valvular heart disease (total; 20,913)

	Valve	Cases	Operation					30-day mortality				Hospital mortality		Redo			
			Mechanical	Bioprosthesis	Ross procedure	Repair	With CABG	Hospital		After discharge				30-day mortality			Hospital mortality
								Replace	Repair	Replace	Repair	Replace	Repair	Cases	Hospital	After discharge	
Isolated	A	9,688	2,219	7,074	3	392	2,316	189 (2.0)	6 (1.5)	9 (0.1)	0	275 (3.0)	6 (1.5)	365	20 (5.5)	0	34 (9.3)
	M	4,617	721	847	0	3,049	773	45 (2.9)	23 (0.8)	1 (0.1)	2 (0.1)	71 (4.5)	41 (1.3)	356	14 (3.9)	0	24 (6.7)
	T	312	9	92		211	42	5 (5.0)	2 (0.9)	0	0	8 (7.9)	5 (2.4)	66	2 (3.0)	0	6 (9.1)
	P	18	0	15		3	1	0	0	0	0	0	0	10	0	0	0
A + M	A	1,380	444	882	0	54	215	65	(4.7)	0		96	(7.0)	100			
	M		303	383	0	694									8 (8.0)	0	11 (11.0)
A + T	A	400	110	281	1	8	45	15	(3.8)	0		29	(7.3)	55			
	T		3	23	0	374									2 (3.6)	0	5 (9.1)
M + T	M	3,388	634	925		1,829	294	65	(1.9)	0		91	(2.7)	274			
	T		6	50		3,332									14 (5.1)	0	26 (9.5)
A + M + T	A	1,040	321	689	0	30	117	37	(3.6)	0		51	(4.9)	76			
	M		262	348	0	430									4 (5.3)	0	5 (6.6)
	T		0	9	1	1,030											
Others		70	18	38	0	16	5	3	(4.3)	0		3	(4.3)	7	1 (14.3)	0	1 (14.3)
Total		20,913	5,050	11,656	5	11,452	3,808	455	(2.2)	12		676	(3.2)	1,309	65 (5.0)	0	112 (8.6)

Number of redo cases is included in total case number of 18,713

Values in parenthesis represent mortality %

*CABG* coronary artery bypass grafting, *A* aortic valve, *M* mitral valve, *T* tricuspid valve, *P* pulmonary valve

**Table:** (2) Ischemic heart disease (total, (A) + (B) + (C); 16,752) *(A) Isolated CABG (total; (a) + (b); 15,462)* (a-1) On-pump arrest CABG (total; 3,749)

	Primary, elective				Primary, emergency				Redo, elective				Redo, emergency				Arterial graft only	Artery graft+SVG	SVG only	Others
	Cases	30-day mortality		Hospital mortality	Cases	30-day mortality		Hospital mortality	Cases	30-day mortality		Hospital mortality	Cases	30-day mortality		Hospital mortality				
		Hospital	After discharge			Hospital	After discharge			Hospital	After discharge			Hospital	After discharge					
1VD	79	0	0	1 (1.3)	20	2 (10.0)	0	2 (10.0)	2	0	0	0	0	0	0	0	66	6	29	0
2VD	454	4 (0.9)	0	5 (1.1)	44	2 (4.5)	0	2 (4.5)	10	1 (10.0)	0	1 (10.0)	4	2 (50.0)	0	2 (50.0)	111	367	34	0
3VD	1,648	13 (0.8)	0	17 (1.0)	203	12 (5.9)	0	14 (6.9)	12	0	0	0	0	0	0	0	132	1,691	40	0
LMT	1,003	16 (1.6)	0	20 (2.0)	255	13 (5.1)	0	17 (6.7)	14	2 (14.3)	0	2 (14.3)	1	1 (100.0)	0	1 (100.0)	146	1,080	47	0
Uncertain						0														
Total	3,184	33 (1.0)	0	43 (1.4)	522	29 (5.6)		35 (6.7)	38	3 (7.9)		3 (7.9)	5	3 (60.0)		3 (60.0)	455	3,144	150	0
Kawasaki	10	0	0	0	1	0	0	0	0	0	0	0	0	0	0	0	7	4	0	0
Hemodialysis	193	7 (3.6)	0	8 (4.1)	40	9 (22.5)	0	10 (25.0)	4	0	0	0	2	0	0	0	13	217	9	0

Values in parenthesis represent mortality %

LMT includes LMT alone or LMT with other branch diseases. *CABG* coronary artery bypass grafting, *1VD* one-vessel disease, *2VD* two-vessel disease, *3VD* three-vessel disease, *LMT* left main trunk, *SVG* saphenous vein graft

**Table:** (a-2) On-pump beating CABG (total; 2,214)

	Primary, elective				Primary, emergency				Redo, elective				Redo, emergency				Arterial graft only	Artery graft+SVG	SVG only	Others
	Cases	30-day mortality		Hospital mortality	Cases	30-day mortality		Hospital mortality	Cases	30-day mortality		Hospital mortality	Cases	30-day mortality		Hospital mortality				
		Hospital	After discharge			Hospital	After discharge			Hospital	After discharge			Hospital	After discharge					
1VD	23	0	0	1 (4.3)	14	0	0	1 (7.1)	4	0	0	0	4	1 (25.0)	0	2 (50.0)	22	5	18	0
2VD	235	2 (0.9)	0	6 (2.6)	65	7 (10.8)	0	7 (10.8)	11	1 (9.1)	0	1 (9.1)	3	0	0	0	65	223	25	1
3VD	805	8 (1.0)	1 (0.1)	16 (2.0)	211	20 (9.5)	0	28 (13.3)	12	0	0	1 (8.3)	1	0	0	0	107	889	33	0
LMT	550	4 (0.7)	0	9 (1.6)	264	27 (10.2)	1 (0.4)	36 (13.6)	10	0	0	0	2	0	0	0	139	639	48	0
Total	1,613	14 (0.9)		32 (2.0)	554	54 (9.7)		72 (13.0)	37	1 (2.7)		2 (5.4)	10	1 (10.0)		2 (20.0)	333	1,756	124	1
Kawasaki	1	0	0	0	1	0	0	0	0	0	0	0	0	0	0	0	1	1	0	0
Hemodialysis	158	2 (1.3)	0	8 (5.1)	46	5 (10.9)	0	5 (10.9)	3	0	0	1 (33.3)	3	0	0	0	18	176	16	0

Values in parenthesis represent mortality %

LMT includes LMT alone or LMT with other branch diseases. *CABG* coronary artery bypass grafting, *1VD* one-vessel disease, *2VD* two-vessel disease, *3VD* three-vessel disease, *LMT* left main trunk, *SVG* saphenous vein graft

**Table:** (b) Off-pump CABG (total; 9,499) (The present section also includes cases of planned off-pump CABG in which, during surgery, the change is made to an on-pump CABG or on-pump beating-heart procedure)

	Primary, elective				Primary, emergency				Redo, elective				Redo, emergency				Arterial graft only	Artery graft+SVG	SVG only	Others
	Cases	30-day mortality		Hospital mortality	Cases	30-day mortality		Hospital mortality	Cases	30-day mortality		Hospital mortality	Cases	30-day mortality		Hospital mortality				
		Hospital	After discharge			Hospital	After discharge			Hospital	After discharge			Hospital	After discharge					
1VD	582	1 (0.2)	0	6 (1.0)	67	2 (3.0)	0	3 (4.5)	40	0	1	0	8	0	0	0	590	48	59	0
2VD	1,484	6 (0.4)	0	12 (0.8)	135	3 (2.2)	0	5 (3.7)	18	2 (11.1)	0	2 (11.1)	5	0	0	0	630	961	41	0
3VD	3,645	11 (0.3)	1 (0.03)	31 (0.9)	390	14 (3.6)	0	21 (5.4)	22	0	0	0	3	1 (33.3)	0	1 (33.3)	772	3,221	61	2
LMT	2,496	14 (0.6)	0	24 (1.0)	574	23 (4.0)	0	31 (5.4)	24	0	0	0	6	1 (16.7)	0	1 (16.7)	855	2,161	75	0
Total	8,207	32 (0.4)	1 (0.01)	73 (0.9)	1,166	42 (3.6)		60 (5.1)	104	2 (1.9)		2 (1.9)	22	2 (9.1)		2 (9.1)	2,847	6,391	236	2
Kawasaki	6	0	0	0	1	0	0	0	1	0	0	0	0	0	0	0	7	1	0	0
Hemodialysis	659	7 (1.1)	0	15 (2.3)	94	10 (10.6)	0	17 (18.1)	10	0	0	0	0	0	0	0	163	581	19	0

Values in parenthesis represent mortality %

LMT includes LMT alone or LMT with other branch diseases. *CABG* coronary artery bypass grafting, *1VD* one-vessel disease, *2VD* two-vessel disease, *3VD* three-vessel disease, *LMT* left main trunk, *SVG* saphenous vein graft

**Table:** (c) Includes cases of conversion, during surgery, from off-pump CABG to on-pump CABG or on-pump beating-heart CABG (total; 197)

	Primary, elective				Primary, emergency				Redo, elective				Redo, emergency			
	Cases	30-day mortality		Hospital mortality	Cases	30-day mortality		Hospital mortality	Cases	30-day mortality		Hospital mortality	Cases	30-day mortality		Hospital mortality
		Hospital	After discharge			Hospital	After discharge			Hospital	After discharge			Hospital	After discharge	
A conversion to on-pump CABG arrest heart	33	1 (3.0)	0	1 (3.0)	5	0	0	0	0	0	0	0	0	0		0
A conversion to on-pump beating-heart CABG	117	5 (4.3)	0	6 (5.1)	41	3 (7.3)	0	3 (7.3)	1	0	0	0	0	0		0
Total	150	7 (4.7)	0	7 (4.7)	46	3 (6.5)		3 (6.5)	1	0	0	0	0	0	0	0
Hemodialysis	13	1 (7.7)	0	1 (7.7)	6	2 (33.3)		2 (33.3)	0	0	0	0	0	0	0	0

Values in parenthesis represent mortality %

*CABG* coronary artery bypass grafting

**Table:** *(B) Operation for complications of MI (total; 1,274)*

	Chronic				Acute				Concomitant operation		
	Cases	30-day mortality		Hospital mortality	Cases	30-day mortality		Hospital mortality			
		Hospital	After discharge			Hospital	After discharge		CABG	MVP	MVR
Infarctectomy or aneurysmectomy	350	18 (5.1)	0	29 (8.3)	26	2 (7.7)	0	3 (11.5)	263	107	15
VSP closure	49	4 (8.2)	0	7 (14.3)	240	53 (22.1)	1	64 (26.7)	89	7	10
Cardiac rupture	14	1 (7.1)	0	1 (7.1)	224	75 (33.5)	0	85 (37.9)	40	2	4
Mitral regurgitation											
1) Papillary muscle rupture	11	0	0	0	35	9 (25.7)	0	11 (31.4)	18	10	23
2) Ischemic	285	18 (6.3)	0	30 (10.5)	28	4 (14.3)	0	4 (14.3)	262	225	48
Others	5	0	0	0	7	1 (14.3)	0	1 (14.3)	2	0	0
Total	714	41 (5.7)	0	67 (9.4)	560	144 (25.7)	1	168 (30.0)	674	351	100

Values in parenthesis represent mortality %

Acute, within 2 weeks from the onset of myocardial infarction

*MI* myocardial infarction, *CABG* coronary artery bypass grafting, *MVP* mitral valve repair, *MVR* mitral valve replacement, *VSP* ventricular septal perforation

**Table:** *(C) TMLR (total; 16)*

	Cases	30-day mortality		Hospital mortality
		Hospital	After discharge	
Isolated	14	2 (14.29)	0	2 (14.29)
With CABG	2	0	0	0
Total	16	2 (12.5)	0	2 (12.5)

Values in parenthesis represent mortality %

*TMLR* transmyocardial laser revascularization

**Table:** (3) Operation for arrhythmia (total; 4,183)

	Cases	30-day mortality		Hospital mortality	Concomitant operation						
					Isolated	Congenital	Valve	IHD	Others	Multiple combination	
		Hospital	After discharge							2 Categories	3 Categories
Maze	3,935	42 (1.1)	0	62 (1.6)	15	197	3,471	493	198	462	24
For WPW	0	0	0	0	0	0	0	0	0	0	0
For ventricular tachyarrhythmia	44	1 (2.3)	0	2 (4.5)	2	3	13	27	7	8	0
Others	204	10 (4.9)	0	13 (6.4)	3	39	141	43	18	38	1
Total	4,183	53 (1.3)	0	77 (1.8)	20	239	3,625	563	223	508	25

Values in parenthesis represent mortality %. Except for 20 isolated cases, all remaining 4,163 cases are doubly allocated, one for this subgroup and the other for the subgroup corresponding to the concomitant operations

*WPW* Wolff–Parkinson–White syndrome, *IHD* ischemic heart disease

**Table:** (4) Operation for constrictive pericarditis (total; 195)

	CPB (+)				CPB (−)			
	Cases	30-day mortality		Hospital mortality	Cases	30-day mortality		Hospital mortality
		Hospital	After discharge			Hospital	96	
Total	96	9 (9.4)	0	18 (18.8)	99	5 (5.1)	0	9 (9.1)

Values in parenthesis represent mortality %

*CPB* cardiopulmonary bypass

**Table:** (5) Cardiac tumor (total; 628)

	Cases	30-day mortality		Hospital mortality	Concomitant operation			
		Hospital	After discharge		AVR	MVR	CABG	Others
Benign tumor	553	6 (1.1)		8 (1.4)	14	7	25	58
Cardiac myxoma	405	2 (0.5)	0	3 (0.7)	4	3	17	41
Papillary fibroelastoma	47	0	0	0	6	2	0	7
Rhabdomyoma	4	0	0	0	0	0	0	1
Others	97	4 (4.1)	0	5 (5.2)	4	2	8	9
Malignant tumor	75	2 (2.7)	1 (1.3)	6 (8.0)	0	0	2	8
Primary	43	2 (4.7)	1 (2.3)	5 (11.6)	0	0	2	5
Metastatic	32	0	0	1 (3.1)	0	0	0	3

Values in parenthesis represent mortality %

*AVR* aortic valve replacement, *MVR* mitral valve replacement, *CABG* coronary artery bypass grafting

**Table:** (6) HOCM and DCM (total; 217)

	Cases	30-day mortality		Hospital mortality	Concomitant operation			
		Hospital	After discharge		AVR	MVR	MVP	CABG
Myectomy	133	4 (3.0)	0	16 (12.0)	67	21	12	12
Myotomy	6	0	0	0	1	0	3	0
No-resection	38	1 (2.6)	0	7 (18.4)	8	21	13	3
Volume reduction surgery of the left ventricle	40	3 (7.5)	0	4 (10.0)	1	8	24	7
Total	217	8 (3.7)	0	27 (12.4)	77	50	52	22

Values in parenthesis represent mortality %

*HOCM* hypertrophic obstructive cardiomyopathy, *DCM* dilated cardiomyopathy, *AVR* aortic valve replacement, *MVR* mitral valve replacement, *MVP* mitral valve repair, *CABG* coronary artery bypass grafting

**Table:** (7) Other open-heart operation (total; 452)

	Cases	30-day mortality		Hospital mortality
		Hospital	After discharge	
Total	452	38 (8.4)	0	53 (11.7)

Values in parenthesis represent mortality %

**Table:** **Table 3** Thoracic aortic aneurysm (total; 14,944) (1) Dissection (total; 6,266)

Replaced site	Stanford type																								
	Acute								Chronic								Concomitant operation					Redo			
	A				B				A				B												
	Cases	30-day mortality		Hospital mortality	Cases	30-day mortality		Hospital mortality	Cases	30-day mortality		Hospital mortality	Cases	30-day mortality		Hospital mortality	AVP	AVR	MVP	MVR	CABG	Cases	30-day mortality		Hospital mortality
		Hospital	After discharge			Hospital	After discharge			Hospital	After discharge			Hospital	After discharge								Hospital	After discharge	
1. Ascending Ao.	2,447	190 (7.8)	2 (0.1)	232 (9.5)	3	1 (33.3)	0	1 (33.3)	222	5 (2.3)	0	6 (2.7)	8	1 (12.5)	0	1 (12.5)	240	134	17	10	125	49	8 (16.3)	0	8 (16.3)
2. Aortic Root	149	28 (18.8)	0	33 (22.1)	0	0	0	0	68	7 (10.3)	0	9 (13.2)	4	0	0	0	21	118	5	1	42	48	9 (18.8)	0	11 (22.9)
3. Ascending Ao.+Arch	1,340	116 (8.7)	2 (0.15)	150 (11.2)	16	3 (18.8)	0	3 (18.8)	302	8 (2.6)	1 (0.3)	18 (6.0)	107	4 (3.7)	0	9 (8.4)	112	61	10	3	87	90	4 (4.4)	0	5 (5.6)
4. Arch+Descending Ao.	30	4 (13.3)	0	4 (13.3)	10	4 (40.0)	0	5 (50.0)	21	1 (4.8)	0	1 (4.8)	79	6 (7.6)	0	9 (11.4)	0	1	0	0	5	9	1 (11.1)	0	2 (22.2)
5. Aortic Root+Asc. Ao.+Arch	96	21 (21.9)	1 (1.0)	22 (22.9)	0	0	0	0	27	0	0	0 (0.0)	4	0	0	0	18	51	0	1	13	10	0	0	0
6. Descending Ao.	11	0	0	1 (9.1)	52	8 (15.4)	0	11 (21.2)	77	4 (5.2)	0	6 (7.8)	217	8 (3.7)	0	14 (6.5)	1	4	0	0	4	38	6 (15.8)	0	7 (18.4)
7. Thoracoabdominal Ao.	5	0	0	0	12	1 (8.3)	0	2 (16.7)	49	3 (6.1)	0	3 (6.1)	151	13 (8.6)	0	16 (10.6)	0	0	0	0	0	43	4 (9.3)	0	4 (9.3)
8. Extra-anatomical bypass	11	0	0	1 (9.1)	19	1 (5.3)	0	2 (10.5)	4	0	0	0 (0.0)	1	0	0	0	0	0	0	0	0	0	0	0	0
9. Stent graft^*a^	97	1 (1.0)	0	1 (1.0)	120	6 (5.0)	0	8 (6.7)	100	3 (3.0)	0	5 (5.0)	518	8 (1.5)	0	15 (2.9)	5	3	0	0	5	99	2 (2.0)	0	3 (3.0)
1) TEVARl^*b^	35	1 (2.9)	0	1 (2.9)	110	6 (5.5)	0	8 (7.3)	86	3 (3.5)	0	5 (5.8)	492	7 (1.4)	0	13 (2.6)	2	0	0	0	0	97	2 (2.1)	0	3 (3.1)
2) Open stent	62	0	0	0	10	0	0	0	14	0	0	0	23	1 (4.3)	0	2 (8.7)	3	3	0	0	5	2	0	0	0
a) With total arch^*c^	3	0	0	0	4	0	0	0	1	0	0	0	4	0	0	0	1	0	0	0	0	1	0	0	0
b) Without total arch^*d^	59	0	0	0	6	0	0	0	13	0	0	0	19	1 (5.3)	0	2 (10.5)	2	3	0	0	5	1	0	0	0
3) Unspecified	0	0	0	0	0	0	0	0	0	0	0	0	3	0	0	0	0	0	0	0	0	0	0	0	0
Total	4,186	360	5 (0.12)	444 (10.6)	232	24 (10.3)	0	30 (12.9)	865	36 (4.2)	1 (0.1)	52 (6.0)	983	34 (3.5)	0	42 (4.3)	339	326	32	16	294	335	29 (8.7)	0	35 (10.4)

Values in parenthesis represent mortality %

*Ao* aorta, *AVP* aortic valve repair, *AVR* aortic valve replacement, *MVP* mitral valve repair, *MVR* mitral valve replacement, *CABG*. coronary artery bypass grafting, *TEVAR* thoracic endovascular aortic(aneurysm) repair

Acute, within 2 weeks from the onset

*a = *b + *c + *d + unspecified

**Table:** (2) Non-dissection (total; 8,678)

Replaced site	Unruptured				Ruptured				Concomitant operation					Redo				CPB(−)			
	Cases	30-day mortality		Hospital mortality	Cases	30-day mortality		Hospital mortality	AVP	AVR	MVP	MVR	CABG	Cases	30-day mortality		Hospital mortality	Cases	30-day mortality		Hospital mortality
		Hospital	After discharge			Hospital	After discharge								Hospital	After discharge			Hospital	After discharge	
1. Ascending Ao.	1,184	19 (1.6)	1 (0.1)	38 (3.2)	50	7 (12.9)	0	10 (20.0)	111	755	66	35	159	88	3 (3.4)	0	11 (12.5)	5	0	0	1 (20.0)
2. Aortic Root	824	16 (1.9)	0	29 (3.5)	31	4 (30.8)	0	9 (29.0)	187	448	47	9	89	109	13 (11.9)	0	24 (22.0)	14	0	0	0
3. Ascending Ao.+Arch	2,106	47 (2.2)	3	87 (4.1)	168	32 (14.8)	0	44 (26.2)	26	202	28	2	338	89	8 (9.0)	0	11 (12.4)	29	0	0	1 (3.4)
4. Arch+Descending Ao.	111	10 (9.0)	0	13 (11.7)	7	2 (34.3)	0	2 (28.6)	0	2	0	0	8	9	3 (33.3)	0	3 (33.3)	9	0	0	0
5. Aortic Root+Asc.Ao.+Arch	93	5 (5.4)	0	9 (9.7)	2	1 (50.0)	0	1 (50.0)	12	47	3	1	9	18	3 (16.7)	0	4 (22.2)	3	0	0	0
6. Descending Ao.	323	15 (4.6)	0	23 (7.1)	85	18 (19.7)	0	21 (24.7)	4	1	0	0	10	33	13 (39.4)	0	14 (42.4)	15	0	0	0
7. Thoracoabdominal Ao.	368	23 (6.3)	0	33 (9.0)	43	8 (24.3)	0	10 (23.3)	0	0	0	0	4	40	4 (10.0)	0	5 (12.5)	15	0	0	0
8. Extra-anatomical bypass	44	2 (4.5)	0	3 (6.8)	3	2 (66.7)	0	2 (66.7)	0	0	0	0	4	1	0	0	0	9	1 (11.1)	0	2 (22.2)
9. Stent graft*^a^	2,889	56 (1.9)	1 (0.03)	80 (2.8)	347	40 (12.9)	1 (0.3)	56 (16.1)	6	8	0	0	20	193	10 (5.2)	0	14 (7.3)	1,212	37 (3.1)	0	50 (4.1)
1) TEVARl*^b^	2,671	47 (1.8)	1 (0.04)	66 (2.5)	335	40 (13.7)	1 (0.3)	54 (16.1)	6	2	0	0	8	184	10 (5.4)	0	14 (7.6)	1,152	37 (3.2)	0	48 (4.2)
2) Open stent	216	9 (4.2)	0	14 (6.5)	10	0	0	2 (20.0)	0	6	0	0	12	9	1 (11.1)	0	0	56	0	0	2 (3.6)
a) With total arch*^c^	78	5 (6.4)	0	7 (9.0)	4	0	0	0	0	0	0	0	3	4	0	0	0	31	0	0	1 (3.2)
b) Without total arch*^d^	138	4 (2.9)	0	7 (5.1)	6	0	0	2 (33.3)	0	6	0	0	9	5	0	0	0	25	0	0	1 (4.0)
3) Unspecified	2	0		0	2	0	0	0	0	0	0	0	0	0	0	0	0	0	0	0	0
Total	7,942	193 (2.4)	5 (0.06)	315 (4.0)	736	114 (15.5)	1 (0.1)	155 (22.2)	346	1,463	144	47	641	580	57 (9.8)	0	86 (14.8)	1,311	38 (3.0)	0	54 (4.1)

Values in parenthesis represent mortality %

*Ao* aorta, *AVP* aortic valve repair, *AVR* aortic valve replacement, *MVP* mitral valve repair, *MVR* mitral valve replacement, *CABG* coronary artery bypass grafting, *TEVAR* thoracic endovascular aortic (aneurysm) repair

*a = *b + *c + *d + unspecified

**Table:** **Table 4** Pulmonary thromboembolism (total; 121)

	Cases	30-day mortality		Hospital mortality
		Hospital	After discharge	
Acute	65	11 (16.9)	0	13 (20.0)
Chronic	56	0	0	1 (1.8)
Total	121	11 (9.1)	0	14 (11.6)

Values in parenthesis represent mortality %

**Table:** **Table 5** Assisted circulation (total; 1,875)

Sites	VAD									Heart–Lung assist					
	Device			Results						Method		Results			
	Centrifugal	VAS	Others	Not weaned			Weaned			PCPS	Others	Not weaned		Weaned	
				On going	Death	Transplant	Alive	Deaths	Transplant			Deaths	Transplant	Deaths	Alive
Post-cardiotomy															
Left	4	6	3	2	10 (76.9)	0	0	1 (10.0)	0						
Right	3	0	0	0	1 (33.3)	0	2	0	0						
Biventricle															
Right	6	1	0	2	5 (71.4)	0	0	0	0	517	70	268 (51.8)	0	99 (19.1)	220
Left	2	5	0												
Congestive heart failure															
Left	23	38	57	70	32 (27.1)	0	8	5 (4.2)	3						
Right	0	0	0	0	0	0	0	0	0						
Biventricle															
Right	23	6	0	5	18 (62.1)	0	5	1 (9.1)	0	888	68	439 (49.4)	1	139 (15.7)	376
Left	8	19	2												
Respiratory failure										75	51	40 (53.3)	0	15 (20.0)	71
Total	69	75	62	79	66 (32.0)	0	15	7 (3.4)	3	1,480	189	747 (50.4)	2	253 (17.1)	667

Values in parenthesis represent mortality %

*VAD* ventricular assist device, *VAS* ventricular assist system, *PCPS* percutaneous cardiopulmonary support

**Table:** **Table 6** Heart transplantation (total; 28)

	Cases	30-day mortality		Hospital mortality
		Hospital	After discharge	
Heart transplantation	28	1 (3.6)	0	2 (7.1)
Heart and lung transplantation	0	0	0	0
Total	28	1 (3.6)	0	2 (7.1)

Values in parenthesis represent mortality %

**Table:** **Table 7** Pacemaker + ICD (total; 6,971)

	Pacemaker			ICD	
	V	A-V	CRT	CRTD	ICD
Initial	942	2,715	117	299	342
Exchange	727	1,439	32	113	197
Unclear	2	46	0	0	0
Total	1,671	4,200	149	412	539

*ICD* implantable cardioverter-defibrillator, *CRTD* cardiac resynchronization therapy device with incorporated ICD device

### (B) General thoracic surgery

The total number of operations reported in 2012 in general thoracic surgery has reached 72,899, which means an increase of 3,676 cases compared with the number of operations in 2011. This is largely owing to the steady increase in lung cancer surgery (31,301; 2009, 32,801; 2010, 33,878:2011, 35,667:2012).

Surgery for lung cancer consists more than 49 % of all the general thoracic surgery. Among lung cancer subtypes, adenocarcinoma comprises an overwhelming percentage of 69.4 % of the total lung cancer surgery, followed by squamous cell carcinoma of 19.0 %. Limited resection by wedge resection or segmentectomy was performed in 6,789 lung cancer patients, which is 19.0 % of the entire cases. Lobectomy was performed 26,079 patients, which is 73.1 % of the entire cases. Sleeve lobectomy was done in 429 patients. Pneumonectomy was done in 571 patients, which is only 1.6 % of the entire cases. VATS (video-assisted thoracic surgery) procedure is performed in 65.6 % among the total lung cancer surgeries in 2012. 123 patients died within 30 days after lung cancer surgery (30-day mortality rate 0.34 %), and 248 patients died without discharge (Hospital mortality rate 0.70 %). 30-day mortality rate in regard to procedures is 0.26 % in segmentectomy, 0.31 % in lobectomy, and 2.45 % in pneumonectomy.

Interstitial pneumonia was the leading cause of death after lung cancer surgery, followed by pneumonia, cardiovascular event, bronchopleural fistula, and respiratory failure.

7,403 patients with metastatic pulmonary tumor were operated in 2012 with steady increase similar to lung cancer surgery (6,248:2009, 6,748:2010, 7,210:2011). VATS was adopted in 5,828 cases, which comprises 78.7 % of the entire cases. Colorectal cancer was by far the leading primary malignancy indicated for resection of metastatic tumors.

73 tracheal tumors were operated in 2012. Adenoid cystic carcinoma and squamous cell carcinoma were frequent primary tracheal tumor.

409 tumors of pleural origin were operated in 2012. Diffuse malignant pleural mesothelioma was the most frequent histology. Extrapleural pneumonectomy was the most frequently chosen operative method (135 cases) with a hospital death of 6.7 %.

752 chest wall tumors were resected in 2012.

4,671 mediastinal tumors were operated in 2012. There were 2,151 thymic epithelial tumors (1,842 thymomas, 271 thymic carcinomas, and 38 thymic carcinoid), followed by 906 congenital cysts, 495 neurogenic tumors, and 231 germ cell tumors. 2,425 cases (51.9 %) were resected by VATS.

Thymectomy for myasthenia gravis was done in 446 patients, and 302 among them were associated with thymoma, indicating that thymectomy for non-thymomatous myasthenia gravis was done only in 144 patients. Advancement in medical control of myasthenia gravis by immunosuppressants might reduce indication of extended thymectomy for non-thymomatous myasthenia gravis. This possibility should be further examined.

2,250 operations for empyema were reported in 2012. There were 1,710 patients (76 %) with acute empyema and 540 patients with chronic empyema. Bronchopleural fistula was associated in 348 patients (20.4 %) with acute empyema and 274 patients (50.7 %) with chronic empyema. It should be noted that hospital mortality was as high as 12.1 % in patients of acute empyema with fistula.

14,410 operations for pneumothorax were reported in 2012. 13,555 operations (94.1 %) were performed by VATS, similarly to cases in 2011.

44 lung transplantations were reported in 2012. The number of the brain-dead donors is slightly increasing after revision of the law on organ transplantation.

The number of lung transplantation operation is still small compared to those in North America and European countries because of shortage of donors, but the number of brain-dead donors is increasing slowly in Japan after revision of the law of organ transplantation in 2010.

**Table:** **Table 1** Total entry cases of general thoracic surgery during 2012

	Cases	%
Benign pulmonary tumor	863	1.2
Primary lung cancer	35,667	48.9
Other primary malignant pulmonary tumor	348	0.5
Metastatic pulmonary tumor	7,403	10.2
Tracheal tumor	73	0.1
Mesothelioma	409	0.6
Chest wall tumor	752	1.0
Mediastinal tumor	4,671	6.4
Thymectomy for MG without thymoma	302	0.4
Inflammatory pulmonary disease	3,405	4.7
Empyema	2,250	3.1
Bullous disease excluding pneumothorax	569	0.8
Pneumothorax	14,410	19.8
Chest wall deformity	415	0.6
Diaphragmatic hernia including traumatic	112	0.2
Chest trauma excluding diaphragmatic hernia	393	0.5
Lung transplantation	44	0.1
Others	813	1.1
Total	72,899	100.0

**Table:** **Table 2** 1. Benign pulmonary tumor

	Cases	30-day mortality		Hospital mortality	By VATS
		Hospital	After discharge		
Hamartoma	421	0	0	0	371
Sclerosing hemangioma	104	0	0	0	84
Papilloma	9	0	0	0	7
Mucous gland adenoma bronchial	5	0	0	0	5
Fibroma	34	0	0	0	26
Lipoma	5	0	0	0	4
Neurogenic tumor	13	0	0	0	10
Clear cell tumor	1	0	0	0	1
Leiomyoma	13	0	0	0	8
Chondroma	3	0	0	0	3
Inflammatory myofibroblastic tumor	3	0	0	0	2
Pseudolymphoma	23	0	0	0	17
Histiocytosis	11	0	0	0	8
Teratoma	4	0	0	0	2
Others	214	0	0	0	171
Total	863	0	0	0	719

Values in parenthesis represent mortality %

**Table:** **Table 3** 2. Primary malignant pulmonary tumor

			Cases	30-day mortality				Hospital mortality		By VATS
				Hospital		After discharge				
2. Primary malignant pulmonary tumor			36,015	103 (0.3)		15 (0.04)		243 (0.7)		
Lung cancer			35,667	112 (0.3)		11 (0.03)		248 (0.7)		23,411
Adenocarcinoma			24,749	48 (0.2)		5 (0.02)		84 (0.3)		
Squamous cell carcinoma			7,114	47 (0.7)		4 (0.06)		122 (1.7)		
Large cell carcinoma			917	3 (0.3)		0		14 (1.5)		
* (LCNEC)*			*471*	*2 (0.4)*		*0*		*6 (1.3)*		
Small cell carcinoma			591	2 (0.3)		0		3 (0.5)		
Adenosquamous carcinoma			612	6 (1.0)		1		11 (1.8)		
Carcinoma with pleomorphic, sarcomatoid or sarcomatous elements			473	2 (0.4)		1		6 (1.3)		
Carcinoid			195	0		1 (0.5)		0		
Carcinomas of salivary gland type			29	0		0		0		
Unclassified			73	0		0		0		
Multiple lung cancer			803	1 (0.1)		0		4 (0.5)		
Others			110	3 (2.7)		0		4 (3.6)		
Unclear			1	0						
Wedge resection			4,952	11 (0.2)		0		18 (0.4)		4,135
Segmental excision			3,780	8 (0.2)		2 (0.1)		10 (0.3)		2,654
* (Sleeve segmental excision)*			*16*	*0*		*0*		*0*		*4*
Lobectomy			26,079	72 (0.3)		9 (0.03)		178 (0.7)		16,416
* (Sleeve lobectomy)*			*429*	*4 (0.9)*		*1 (0.2)*		*7 (1.6)*		*29*
Pneumonectomy			571	14 (2.5)		0		30 (5.3)		75
*(Sleeve pneumonectomy)*			*16*	*0*		*0*		*0*		*0*
Other bronchoplasty			10	0		0		0		3
Pleuropneumonectomy			4	0		0		0		1
Others			265	3 (1.1)		0		5 (1.9)		127
Unclear			11	4 (36.4)				7 (63.6)		
Sarcoma			36	0		0		1 (2.8)		
AAH			143	0		0		1 (0.7)		
Others			169	0		0		2 (1.2)		

Values in parenthesis represent mortality %

**Table:** **Table 4** Details of lung cancer operation TNM

	Cases
c-Stage	
Ia	20,510
Ib	7,019
IIa	2,712
IIb	1,783
IIIa	2,493
IIIb	256
IV	414
NA	480
Total	35,667
Sex	
Male	22,103
Female	13,426
NA	138
Total	35,667
Cause of death	
Cardiovascular	25
Pneumonia	52
Pyothorax	8
Bronchopleural fistula	25
Respiratory failure	25
Pulmonary embolism	3
Interstitial pneumonia	71
Brain infarction or bleeding	15
Others	32
Unknown	3
Total	259
p-Stage	
0 (pCR)	157
Ia	17,804
Ib	7,264
IIa	3,011
IIb	2,093
IIIa	3,806
IIIb	306
IV	943
NA	283
Total	35,667
Age	
<20	4
20–29	22
30–39	223
40–49	1,001
50–59	3,630
60–69	12,336
70–79	14,299
80–89	4,100
≥90	50
NA	2
Total	35,667

**Table:** **Table 5** 3. Metastatic pulmonary tumor

	Cases	30-day mortality				Hospital mortality		By VATS
		Hospital		After discharge				
3. Metastatic pulmonary tumor	7,403	3 (0.04)		0		9 (0.1)		5,828
Colorectal	3,639	0		0		3 (0.1)		2,910
Hepatobiliary/Pancreatic	311	0		0		1 (0.3)		240
Uterine	380	1 (0.26)		0		0		302
Mammary	456	0		0		1 (0.2)		386
Ovarian	68	0		0		0		56
Testicular	64	0		0		0		49
Renal	600	0		0		0		500
Skeletal	160	0		0		0		121
Soft tissue	275	0		0		0		214
Otorhinolaryngological	446	0		0		0		338
Pulmonary	384	3 (0.8)		0		3 (0.8)		238
Others	620	1		0		1 (0.2)		474

Values in parenthesis represent mortality %

**Table:** **Table 6** 4. Tracheal tumor

	Cases	30-day mortality		Hospital mortality
		Hospital	After discharge	
4. Tracheal tumor	73	0	0	1 (1.4)
(A) Primary malignant tumor (histological classification)				
Squamous cell carcinoma	4	0	0	0
Adenoid cystic carcinoma	19	0	0	0
Mucoepidermoid carcinoma	1	0	0	0
Others	1	0	0	0
Total	25	0	0	0
(B) Metastatic/invasive malignant tumor e.g. invasion of thyroid cancer	23	0	0	1 (4.3)
(C) Benign tracheal tumor (histological classification)				
Papilloma	1	0	0	0
Adenoma	2	0	0	0
Neurofibroma	2	0	0	0
Chondroma	1	0	0	0
Leiomyoma	0	0	0	0
Others	19	0	0	0
Histology unknown	0	0	0	0
Total	25	0	0	0
Operation				
Sleeve resection with reconstruction	28	0	0	0
Wedge with simple closure	4	0	0	0
Wedge with patch closure	0	0	0	0
Total laryngectomy with tracheostomy	2	0	0	1 (50.0)
Others	38	0	0	0
Unknown	1	0	0	0
Total	73	0	0	1 (1.4)

Values in parenthesis represent mortality %

**Table:** **Table 7** 5. Tumor of pleural origin

	Cases	30-day mortality		Hospital mortality
		Hospital	After discharge	
Histological classification				
Solitary fibrous tumor	130	0	0	0
Diffuse malignant pleural mesothelioma	198	5 (2.5)	0	9 (4.5)
Localized malignant pleural mesothelioma	29	0	0	1
Others	52	0	0	0
Total	409	3 (0.7)	0	10 (2.4)
Operative procedure				
Extrapleural pneumonectomy	135	5 (3.7)	0	9 (6.7)
Total pleurectomy	22	0	0	0
Total parietal pleurectomy	0	0	0	0
Partial pleurectomy	0	0	0	0
Exploratory thoracotomy	0	0	0	0
Others	41	0 (0.0)	0	0
Total	198	5 (2.5)	0	9 (4.5)

Values in parenthesis represent mortality %

**Table:** **Table 8** 6. Chest wall tumor

	Cases	30-day mortality		Hospital mortality	By VATS
		Hospital	After discharge		
Primary malignant tumor	147	1 (0.68)	0	1 (0.7)	26
Metastatic malignant tumor	232	0	0	2 (0.9)	34
Benign tumor	373	0	0	0	199
Total	752	1 (0.1)	0	3 (0.4)	259

Values in parenthesis represent mortality %

**Table:** **Table 9** 7. Mediastinal tumor

	Cases	30-day mortality		Hospital mortality	By VATS
		Hospital	After discharge		
7. Mediastinal tumor	4,671	6 (0.13)	0	7 (0.1)	2,425
Thymoma*	1,842	2 (0.1)	0	2 (0.1)	659
Thymic cancer	271	2 (0.7)	0	3 (1.1)	56
Thymus carcinoid	38	0	0	0	15
Germ cell tumor	231	1 (0.4)	0	1 (0.4)	80
* Benign*	*159*	*0*	*0*	*0*	*72*
* Malignant*	*72*	*1 * (1.4)	*0*	*1 (1.4)*	*8*
Neurogenic tumor	495	0	0	0	412
Congenital cyst	906	0	0	0	759
Goiter	115	0	0	0	28
Lymphatic tumor	210	1 (0.5)	0	1 (0.5)	124
Excision of pleural recurrence of thymoma	93	0	0	0	35
Others	470	0	0	0	257

* Includes those with myasthenia gravis

Values in parenthesis represent mortality %

**Table:** **Table 10** 8. Thymectomy for myasthenia gravis

	Cases	30-day mortality		Hospital mortality	By VATS
		Hospital	After discharge		
8. Thymectomy for myasthenia gravis	446	0	0	0	171
With thymoma	302	1 (0.3)	0	2 (0.7)	81

Values in parenthesis represent mortality %

**Table:** **Table 11** 9. Operation for non-neoplasmic disease (A) Inflammatory pulmonary disease

	Cases	30-day mortality		Hospital mortality	
		Hospital	After discharge		
9. Operation for non-neoplasmic disease	22,367	109 (0.5)	2 (0.01)	181 (0.8)	

	Cases	30-day mortality		Hospital mortality	By VATS
		Hospital	After discharge		
(A) Inflammatory pulmonary disease	3,405	4 (0.1)	0	7 (0.2)	2,556
Tuberculous infection	107	0	0	0	70
Mycobacterial infection	514	0	0	0	395
Fungal infection	439	4 (0.9)	0	5 (1.1)	218
Bronchiectasis	93	0	0	0	60
Tuberculous nodule	351	0	0	0	291
Inflammatory pseudo tumor	1,007	0	0	0	812
Intrapulmonary lymph node	175	0	0	0	161
Others	719	0	0	2 (0.3)	549

Values in parenthesis represent mortality %

**Table:** **Table 12** 9. Operation for non-neoplasmic disease (B) Empyema

	Cases	30-day mortality		Hospital mortality	By VATS
		Hospital	After discharge		
Acute empyema	1,710	29 (1.7)	0	61 (3.6)	1,142
With fistula	348	15 (4.3)	0	42 (12.1)	97
Without fistula	1,349	13 (1.0)	0	18 (1.3)	1,037
Unknown	13	1 (7.7)	0	1 (7.7)	8
Chronic empyema	540	5 (0.9)	0	15 (2.8)	133
With fistula	274	4 (1.5)	0	10 (3.6)	44
Without fistula	263	1 (0.4)	0	5 (1.9)	87
Unknown	3	0	0	0	2
Total	2,250	34 (1.5)	0	76 (3.4)	1,275

Values in parenthesis represent mortality %

**Table:** **Table 13** 9. Operation for non-neoplasmic disease (C) Descending necrotizing mediastinitis

	Cases	30-day mortality		Hospital mortality	By VATS
		Hospital	After discharge		
(C) Descending necrotizing mediastinitis	92	6 (6.5)	0	7 (7.6)	50

Values in parenthesis represent mortality %

**Table:** **Table 14** 9. Operation for non-neoplasmic disease (D) Bullous disease

	Cases	30-day mortality		Hospital mortality	By VATS
		Hospital	After discharge		
(D) Bullous disease	569	0	0	2 (0.4)	443
Emphysematous bulla	438	0	0	2 (0.5)	355
Bronchogenic cyst	74	0	0	0	58
Emphysema with volume reduction surgery	22	0	0	0	16
Others	35	0	0	0	14

Values in parenthesis represent mortality %

*LVRS* lung volume reduction surgery

**Table:** **Table 15** 9. Operation for non-neoplasmic disease (E) Pneumothorax

	Cases	30-day mortality		Hospital mortality	By VATS
		Hospital	After discharge		
(E) Pneumothorax	14,410	37 (0.3)	1 (0.01)	53 (0.4)	13,555
*Spontaneous pneumothorax*					
Operative procedure					
Bullectomy	3,588	3 (0.08)	0	3 (0.08)	3,400
Bullectomy with additional procedure	7,751	1 (0.01)	0	1 (0.01)	7,456
Coverage with artificial material	7,374	1 (0.01)	0	1 (0.01)	7,088
Parietal pleurectomy	34	0	0	0	33
Coverage and parietal pleurectomy	85	0	0	0	81
Others	258	0	0	0	254
Others	475	3 (0.6)	0	0	432
Total	11,814	7 (0.1)	0	4 (0.0)	11,288
*Secondary pneumothorax*					
Associated disease					
COPD	1,977	19 (1.0)	1 (0.1)	25 (1.3)	1,748
Tumorous disease	92	3 (3.3)	0	6 (6.5)	75
Catamenial	153	0	0	0	152
LAM	38	2 (5.26)	0	2	33
Others (excluding pneumothorax by trauma)	333	9 (2.7)	0	16 (4.8)	258
Unknown	3				1
Operative procedure					
Bullectomy	28	5 (17.9)	0	8 (28.6)	344
Bullectomy with additional procedure	1,881	19 (1.0)	1	29 (1.5)	1,656
Coverage with artificial material	1,707	14 (0.8)	1 (0.06)	23 (1.3)	1,506
Parietal pleurectomy	21	0	0	0	20
Coverage and parietal pleurectomy	16	1 (6.3)	0	1 (6.3)	13
Others	137	4 (2.9)	0	5 (3.6)	117
Others	330	9 (2.7)	0	12 (3.6)	264
Unknown	4	0	0	0	3
Total	2,243	33 (1.5)	1 (0.04)	49 (2.2)	2,267

Values in parenthesis represent mortality %

**Table:** **Table 16** 9. Operation for non-neoplasmic disease (F) Chest wall deformity

	Cases	30-day mortality		Hospital mortality
		Hospital	After discharge	
(F) Chest wall deformity	415	0	0	0
Funnel chest	393	0	0	0
Others	22	0	0	0

Values in parenthesis represent mortality %

**Table:** **Table 17** 9. Operation for non-neoplasmic disease (G) Diaphragmatic hernia

	Cases	30-day mortality		Hospital mortality	By VATS
		Hospital	After discharge		
(G) Diaphragmatic hernia	112	3 (2.7)	0	4 (3.6)	34
Congenital	54	1 (1.9)	0	2 (3.7)	11
Traumatic	38	2 (5.3)	0	2 (5.3)	7
Others	30	0	0	0	16

Values in parenthesis represent mortality %

**Table:** **Table 18** 9. Operation for non-neoplasmic disease (H) Chest trauma

	Cases	30-day mortality		Hospital mortality	By VATS
		Hospital	After discharge		
(H) Chest trauma	393	18 (4.6)	1 (0.25)	20 (5.1)	127

Values in parenthesis represent mortality %

**Table:** **Table 19** 9. Operation for non-neoplasmic disease (I) Other respiratory surgery

	Cases	30-day mortality		Hospital mortality	By VATS
		Hospital	After discharge		
(I) Other respiratory surgery	727	7 (1.0)	0	14 (1.9)	392
Arteriovenous malformation*	95	1 (1.1)	0	1 (1.1)	73
Pulmonary sequestration	126	0	0	0	68
Others	506	6 (1.2)	0	13 (2.6)	251

* Includes those with myasthenia gravis

Values in parenthesis represent mortality %

**Table:** **Table 20** 10. Lung transplantation

	Cases	30-day mortality		Hospital mortality
		Hospital	After discharge	
Single lung transplantation from brain-dead donor	16	0	0	1 (6.3)
Bilateral lung transplantation from brain-dead donor	18	0	0	0
Lung transplantation from living donor	10	0	0	0
Total of lung transplantation	44	0	0	1 (2.3)
Donor of living donor lung transplantation	17	0	0	0

Values in parenthesis represent mortality %

**Table:** **Table 21** 11. Video-assisted thoracic surgery

	Cases	30-day mortality		Hospital mortality
		Hospital	After discharge	
11. Video-assisted thoracic surgery	54,616	83 (0.2)	5 (0.01)	141 (0.3)

Values in parenthesis represent mortality %

(Including thoracic sympathectomy 146)

**Table:** **Table 22** 12. Tracheobronchoplasty

	Cases	30-day mortality		Hospital mortality
		Hospital	After discharge	
12. Tracheobronchoplasty	554	3 (0.5)	1 (0.2)	9 (1.6)
Trachea	105	0	0	2 (1.9)
Sleeve resection with reconstruction	58	0	0	0
Wedge with simple closure	29	0	0	1
Wedge with patch closure	1	0	0	0
Total laryngectomy with tracheostomy	2	0	0	1
Others	15	0	0	0
Cardinal reconstruction	7	0	0	0
Sleeve pneumonectomy	16	0	0	1 (6.3)
Sleeve lobectomy	383	3 (0.8)	1 (0.3)	6 (1.6)
Sleeve segmental excision	12	0	0	0
Bronchoplasty without lung resection	12	0	0	0
Others	19	0	0	0

Values in parenthesis represent mortality %

**Table:** **Table 23** 13. Pediatric surgery

	Cases	30-day mortality		Hospital mortality
		Hospital	After discharge	
13. Pediatric surgery	417	1 (0.2)	0	1 (0.2)

Values in parenthesis represent mortality %

**Table:** **Table 24** 14. Combined resection of neighboring organ(s)

	Cases	30-day mortality		Hospital mortality
		Hospital	After discharge	
14. Combined resection of neighboring organ(s)	1,002	5 (0.5)	3 (0.3)	15 (1.5)
(A) Primary lung cancer (organ resected)				
Aorta	9	1 (11.1)	0	1 (11.1)
Superior vena cava	35	0	0	2 (5.7)
Brachycephalic vein	12	1 (8.3)	0	1 (8.3)
Pericardium	171	1 (0.6)	0	3 (1.8)
Pulmonary artery	184	0	0	2 (1.1)
Left atrium	33	1 (3.0)	0	1 (3.0)
Diaphragm	94	0	0	1 (1.1)
Chest wall (including ribs)	494	2 (0.4)	1 (0.2)	12 (2.4)
Vertebra	30	0	0	2 (6.7)
Esophagus	7	0	0	0
Total	1,069	6 (0.6)	1 (0.1)	25 (2.3)
(B) Mediastinal tumor (organ resected)				
Aorta	2	0	0	0
Superior vena cava	55	1 (1.8)	0	1 (1.8)
Brachycephalic vein	85	0	0	1 (1.2)
Pericardium	238	1 (0.4)	0	1 (0.4)
Pulmonary artery	1	0	0	0
Left atrium	0	0	0	0
Diaphragm	24	0	0	0
Chest wall (including ribs)	16	0	0	0
Vertebra	6	0	0	0
Esophagus	3	0	0	0
Lung	290	0	0	0
Total	720	2 (0.3)	0	3 (0.4)

Values in parenthesis represent mortality %

**Table:** **Table 25** 15. Operation of lung cancer invading the chest wall of the apex

	Cases	30-day mortality		Hospital mortality
		Hospital	After discharge	
15. Operation of lung cancer invading the chest wall of the apex	128	0	0	1 (0.01)

Values in parenthesis represent mortality %

Includes tumors invading the anterior apical chest wall and posterior apical chest wall (superior sulcus tumor, so-called Pancoast type)

### (C) Esophageal surgery

During 2012 alone, a total of 12,315 patients with esophageal diseases were registered from 555 institutions (response rate: 95.4 %), affiliated to the Japanese Association for Thoracic Surgery and/or to the Japan Esophageal Society. Among these institutions, 20 or more patients underwent esophageal surgeries within the year of 2012 in 184 institutions (33.2 %), which shows definite shift of esophageal operations to high volume institutions when compared to the data of 2011 (22.9 %) (Table 1) Of 2,281 patients with a benign esophageal disease, 1,141 (50.0 %) patients underwent surgery, and 30 (1.3 %) patients underwent endoscopic resection, while 1,110 (48.7 %) patients did not undergo any surgical treatment. (Table 2) Of 10,034 patients with a malignant esophageal tumor, 7,859 (78.3 %) patients underwent resection, esophagectomy for 6,055 (60.3 %) and endoscopic mucosal resection (EMR) or endoscopic submucosal dissection (ESD) for 1,804 (18.0 %), while 2,175 (21.6 %) patients did not undergo any resection. (Tables 3, 4) The decrease of registered patients with nonsurgically treated benign esophageal diseases is obvious during 2011 and 2012. The patients registered, particularly those undergoing nonsurgical therapy for a malignant esophageal disease, have been increasing since 1990 (Fig. 1).

Among benign esophageal diseases (Table 2), esophageal varices, hiatal hernia, achalasia and esophagitis (including reflux esophagitis) were the most common conditions in Japan. On the other hand, spontaneous rupture of the esophagus, benign esophageal tumors and congenital esophageal atresia were common diseases which were surgically treated. The thoracoscopic and/or laparoscopic procedures have been widely adopted for benign esophageal diseases, in particular achalasia, hiatal hernia and benign tumors. Open surgery was performed in 828 patients with a benign esophageal disease with 30-day mortality in 5 (0.6 %), while thoracoscopic and/or laparoscopic surgery was performed for 233 patients with 0 (0.0 %) of the 30-day mortality The difference in these death rates between open and scopic surgery seems to be related to the conditions requiring open surgery.

The majority of malignant diseases were carcinomas (Table 3). Among esophageal carcinomas, the incidence of squamous cell carcinoma was 91.8 %, while that of adenocarcinomas including Barrett cancer was 5.7 %. The resection rate for patients with a squamous cell carcinoma was 77.6 %, while that for patients with an adenocarcinoma was 91.5 %.

According to location, cancer in the thoracic esophagus was the most common (Table 4). Of the 3,793 patients (37.8 % of total esophageal malignancies) having superficial esophageal cancers within mucosal and submucosal layers, 1,759 (46.3 %) patients underwent esophagectomy, while 1,802 (47.5 %) patients underwent EMR or ESD. The 30-day mortality rate and hospital mortality rate after esophagectomy for patients with a superficial cancer were 0.6 and 1.5 %, respectively. There was no EMR or ESD-related death. Advanced esophageal cancer invading deeper than the submucosal layer was observed in 6,231 (62.1 %) patients. Of the 6,231 patients with advanced esophageal cancer, 4,288 (68.8 %) underwent esophagectomy, with 0.8 % of the 30-day mortality rate, and with 2.4 % of the hospital mortality rate.

Multiple primary cancers were observed in 1,644 (16.4 %) of all the 10,034 patients with esophageal cancer. Synchronous cancer was found in 868 (52.9 %) patients, while metachronous cancer (found before esophageal cancer) was observed in 772 (47.0 %) patients. The stomach is the commonest site for both synchronous and metachronous malignancy followed by head & neck cancer (Table 4).

**Fig. 2:**
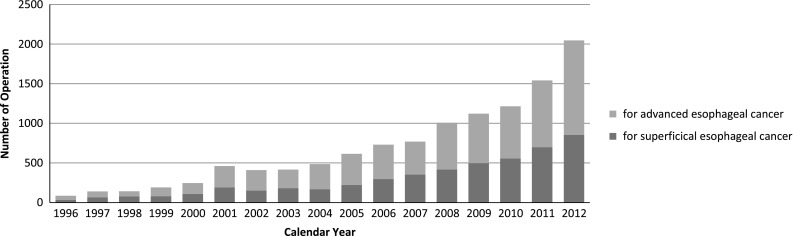
Annual trend of video-assisted esophagectomy for esophageal malignancy

Among esophagectomy procedures, transthoracic esophagectomy through right thoracotomy was the most commonly adopted for patients with a superficial cancer as well as for those with an advanced cancer (Table 5). Transhiatal esophagectomy commonly performed in Western countries was adopted in only 4.4 % of patients having a superficial cancer who underwent esophagectomy, and in 2.0 % of those having an advanced cancer in Japan. The thoracoscopic and/or laparoscopic esophagectomy were adopted for 855 patients (48.6 %) with a superficial cancer, and for 1,193 patients (27.8 %) with an advanced cancer. The number of cases of thoracoscopic and/or laparoscopic surgery for superficial or advanced cancer has been increasing for these several years (Fig. 2).

Combined resection of the neighboring organs during resection of an esophageal cancer was performed in 334 patients (Tables 5, 6). Resection of the aorta together with the esophagectomy was performed in 2 cases. Tracheal and/or bronchial resection combined with esophagectomy was performed in 23 patients, with the 30-day mortality rate at 4.3 % and the hospital mortality rate at 13.0 %. Lung resection combined with esophagectomy was performed in 69 patients, with the 30-day mortality rate at 0 % and the hospital mortality rate at 1.4 %.

Salvage surgery after definitive (chemo-)radiotherapy was performed in 256 patients, with the 30-day mortality rate at 2.7 % and with the hospital mortality rate at 6.3 %. (Table 5).

Lastly, in spite of the efforts of the Committee to cover wider patient populations to this annual survey, the majority of the institutions which responded to the questionnaire were the departments of thoracic or esophageal surgery. It should be noted that larger number of patients with esophageal diseases should have been treated medically and endoscopically. We should continue our effort for complete survey through more active collaboration with the Japan Esophageal Society and other related societies.

**Table:** **Table 1** Distribution of number of esophageal operations in 2012 in each institution

Esophageal surgery			
Number of operations in 2012	Benign esophageal diseases	Malignant esophageal disease	Benign + malignant
*0*	262	116	86
1–4	228	99	99
5–9	34	73	81
10–19	22	98	105
20–29	4	45	48
30–39	3	32	35
40–49	0	30	30
≧50	2	62	71
Total	555	555	555

**Table:** **Table 2** Benign esophageal diseases

	Operation (+)												Endoscopic resection	Operation (−)	Total
	Number of patients			30-day mortality						Hospital mortality					
	Total	Open	T/L*3	Open surgery			T/L*3			Total	Open surgery	T/L*3			
				Total	Hospital	After discharge	Total	Hospital	After discharge						
1. Achalasia	190	129	61	0	0	0	0	0	0	0	0	0		40	230
2. Benign tumor	74	37	37	0	0	0	0	0	0	0	0	0	30	13	117
(1) Leiomyoma	46	21	22	0	0	0	0	0	0	0	0	0	12	9	67
(2) Cyst	7	3	4	0	0	0	0	0	0	0	0	0	0	1	8
(3) Others	21	10	11	0	0	0	0	0	0	0	0	0	18	3	42
(4) Not specified	0	0	0	0	0	0	0	0	0	0	0	0		0	0
3. Diverticulum	28	22	6	0	0	0	0	0	0	0	0	0		14	42
4. Hiatal hernia	382	284	98	0	0	0	0	0	0	0	0	0		136	518
5. Spontaneous rupture of the esophagus	86	79	7	1 (1.3)	1 (1.3)	0	0	0	0	1	1 (1.3)	0		9	95
6. Esophago-tracheal fistula	14	13	1	0	0	0	0	0	0	1	1 (7.7)	0		11	25
7. Congenital esophageal atresia	35	14	1	0	0	0	0	0	0	0 (0.0)	0	0		0	35
8. Congenital esophageal stenosis	12	11	1	0	0	0	0	0	0	0	0	0		9	21
9. Corrosive stricture of the esophagus	11	10	1	0	0	0	0	0	0	0	0	0		18	29
10. Esophagitis, Esophageal ulcer	34	32	2	0	0	0	0	0	0	2 (5.9)	2 (6.3)	0		197	231
11. Esophageal varices	235	167	8	1 (0.6)	1 (0.6)	0	0	0	0	1 (0.4)	1 (0.6)	0		608	843
(1) Laparotomy	31	23	8	0	0	0	0	0	0	0 (0.0)	0	0			31
(2) Others				0		0	0	0	0	0					0
(3) Sclerotherapy				0		0	0	0	0	0				497	497
12. Others	40	30	10	3 (10.0)	3 (10.0)	0	0	0	0	3 (7.5)	3 (10.0)	0		55	95
Total	1,141	828	233	5 (0.6)	5 (0.6)	0	0	0	0	8 (0.7)	8 (1.0)	0	30	1,110	2,281

Values in parenthesis represent mortality %

*T/L* thoracoscopic and/or laparoscopic

**Table:** **Table 3** Malignant esophageal diseases (histologic classification)

	Resection (+)	Resection (−)	Total
Carcinomas	7,823	2,133	9,956
1. Squamous cell carcinoma	7,097	2,044	9,141
2. Basaloid(-squamous) carcinoma	86	11	97
3. Carcinosarcoma	36	2	38
4. Adenocarcinoma in the Barrett’s esophagus	315	26	341
5. Other adenocarcinoma	200	22	222
6. Adenosquamous carcinoma	32	2	34
7. Mucoepidermoid carcinoma	4	0	4
8. Adenoid cystic carcinoma	4	0	4
9. Endocrine cell carcinoma	35	13	48
10. Undifferentiated carcinoma	8	9	17
11. Others	6	4	10
Other malignancies	22	7	29
1. Malignant non-epithelial tumors	7	1	8
2. Malignant melanoma	14	5	19
3. Other malignant tumors	1	1	2
Not specified	14	35	49
Total	7,859	2,175	10,034

Resection: including endoscopic resection

**Table:** **Table 4** Malignant esophageal disease (clinical characteristics)

	Operation (+)					EMR or ESD	Operation (−)	Total
	Cases	30-day mortality			Hospital mortality			
		Total	Hospital	After discharge				
1. Esophageal cancer	6,055	47 (0.8)	45 (0.7)	2 (0.0)	131 (2.2)	1,804	2,175	10,034
Location								0
(1) Cervical esophagus	215	2 (0.9)	2 (0.9)	0	8 (3.7)	78	164	457
(2) Thoracic esophagus	5,022	43 (0.9)	42 (0.8)	1 (0.0)	114 (2.3)	1,506	1,845	8,373
(3) Abdominal esophagus	552	2 (0.4)	1 (0.2)	1 (0.2)	8 (1.4)	78	78	708
(4) Multiple cancers	261	0	0	0	1 (0.4)	118	70	449
(5) Others/not described	5	0	0	0	0	24	18	47
Tumor depth								
(A) Superficial cancer	1,759	11 (0.6)	10 (0.6)	1 (0.1)	26 (1.5)	1,802	232	3,793
(B) Advanced cancer	4,288	36 (0.8)	35 (0.8)	1 (0.0)	104 (2.4)	0	1,943	6,231
(C) Not specified	8					2	0	10
2. Multiple primary cancers	967	8 (0.8)	7 (0.7)	1 (0.1)	20 (2.1)	364	313	1,644
1) Synchronous	558	3 (0.5)	2 (0.4)	1 (0.2)	9 (1.6)	138	172	868
(1) Head and neck	177	1 (0.6)	1 (0.6)	0	2 (1.1)	60	45	282
(2) Stomach	221	0 (0.0)	0	0	4 (1.8)	35	55	311
(3) Others	137	2 (1.5)	0	1 (0.7)	1 (0.7)	27	54	218
(4) Triple cancers	23	1	1 (4.3)	0	2 (8.7)	16	18	57
2) Metachronous	409	5 (1.2)	5 (1.2)	0	11 (2.7)	225	138	772
(1) Head and neck	82	2 (2.4)	2 (2.4)	0	2 (2.4)	93	30	205
(2) Stomach	132	2 (1.5)	2 (1.5)	0	6 (4.5)	51	45	228
(3) Others	171	1 (0.6)	1 (0.6)	0	2 (1.2)	48	43	262
(4) Triple cancers	24	0	0	0	1 (4.2)	32	17	73

Values in parenthesis represent mortality %

*EMR* endoscopic mucosal resection (including endoscopic submucosal dissection)

**Table:** **Table 5** Malignant esophageal disease (surgical procedures)

	Cases	Operation (+)							EMR or ESD
		30-day mortality		Hospital mortality	Thoracoscopic and/or laparoscopic procedure				
		Hospital	After discharge		Cases	30-day mortality		Hospital mortality	
						Hospital	After discharge		
Superficial cancer									
Esophagectomy	*1,759*	*10 (0.6)*	*1 (0.1)*	*26 (1.5)*	*855*	*6 (0.7)*	*0*	*13 (1.5)*	1,802
(1) Transhiatal esophagectomy	76	1 (1.3)	0	2 (2.6)	6	0	0	0	
(2) Transthoracic (rt.) esophagectomy and reconstruction	1,387	9 (0.6)	1 (0.1)	21 (1.5)	735	6 (0.8)	0	12 (1.6)	
(3) Transthoracic (lt.) esophagectomy and reconstruction	54	0	0	1 (1.9)	4	0	0	0	
(4) Cervical esophageal resection and reconstruction	18	0	0	0	3	0	0	0	
(5) Two-stage operation	27	0	0	0	7	0	0	0	
(6) Others	184	0	0	1 (0.5)	76	0	0	0	
(7) Not specified	13	0	0	1	24	0	0	1 (4.2)	
Advanced cancer									
Esophagectomy	*4,288*	*35 (0.8)*	*1 (0.0)*	*104 (2.4)*	*1,193*	*8 (0.7)*	*0*	*21 (1.8)*	0
(1) Transhiatal esophagectomy	84	1 (1.2)	0	6 (7.1)	8	0	0	0	
(2) Transthoracic (rt.) esophagectomy and reconstruction	3,479	22 (0.6)	0	79 (2.3)	1,004	5 (0.5)	0	18 (1.8)	
(3) Transthoracic (lt.) esophagectomy and reconstruction	227	2 (0.9)	1 (0.4)	3 (1.3)	32	0	0	0	
(4) Cervical esophageal resection and reconstruction	120	2 (1.7)	0	4 (3.3)	6	0	0	0	
(5) Two-stage operation	96	2 (2.1)	0	5 (5.2)	18	1 (5.6)	0	1 (5.6)	
(6) Others/not specified	268	5 (1.9)	0	6 (2.2)	116	2 (1.7)	0	2 (1.7)	
(7) Not specified	14	1 (7.1)	0	1 (7.1)	9	0 (0.0)	0	0	
(Depth not specified)	*8*	*0*	*0*	*1 (12.5)*					
Combined resection of other organs	*334*	*2 (0.6)*	*0*	*9 (2.7)*					
(1) Aorta	2	0	0	0					
(2) Trachea, bronchus	23	1 (4.3)	0	3 (13.0)					
(3) Lung	69	0	0	1 (1.4)					
(4) Others	240	1 (0.4)	0	5 (2.1)					
Salvage surgery	*256*	*7 * *(2.7)*	*0*	*16 (6.3)*	*31*	*1 (3.2)*	*0*	*4 (12.9)*	35

Values in parenthesis represent mortality %

**Table:** **Table 6** Mortality after combined resection of the neighboring organs

Year	Esophagectomy			Combined resection											
				Aorta			Tracheobronchus			Lung			Others		
	a	b	c (%)	a	b	c (%)	a	b	c (%)	a	b	c (%)	a	b	c (%)
1996	4,194	120	2.86	7	3	42.86	24	0	0.00	50	2	4.00	78	4	5.13
1997	4,441	127	2.86	1	0	0.00	34	5	14.71	56	1	1.79	94	3	3.19
1998	4,878	136	2.79	4	0	0.00	29	0	0.00	74	1	1.35	128	2	1.56
1999	5,015	116	2.31	5	0	0.00	23	2	8.70	68	0	0.00	122	1	0.82
2000	5,350	81	1.51	2	0	0.00	23	2	8.70	69	0	0.00	96	1	1.04
2001	5,521	110	1.99	1	0	0.00	26	1	3.85	83	3	3.61	99	2	2.02
2002	4,904	66	1.35	3	1	33.33	20	2	10.00	63	0	0.00	63	1	1.59
2003	4,639	45	0.97	0	0	0.00	24	2	8.33	58	0	0.00	88	1	1.14
2004	4,739	64	1.35	2	0	0.00	17	0	0.00	59	5	8.47	119	2	1.68
2005	5,163	52	1.01	1	0	0.00	11	1	9.09	67	1	1.49	73	1	1.37
2006	5,236	63	1.20	0	0	0.00	17	0	0.00	62	2	3.23	122	3	2.46
2007	4,990	60	1.20	0	0	0.00	25	1	4.00	44	1	2.27	138	2	1.45
2008	5,124	63	1.23	0	0	0.00	17	1	5.88	48	1	2.08	185	0	0.00
2009	5,260	63	1.20	0	0	0.00	19	2	10.53	58	2	3.45	211	3	1.42
2010	5,180	45	0.87	2	0	0.00	33	0	0.00	58	0	0.00	245	5	2.04
2011	5,430	38	0.70	4	0	0.00	26	0	0.00	41	0	0.00	179	5	2.79
2012	6,055	47	0.78	2	0	0.00	23	1	4.35	69	0	0.00	240	1	0.42
Total	86,119	1,040	1.21	26	4	15.38	273	16	5.86	753	16	2.12	1,220	23	1.89

*a* number of patients who underwent the operation, *b* number of patients died within 30 days after operation, *c* % ratio of b/a, i.e., direct operative mortality

**Fig. 1:**
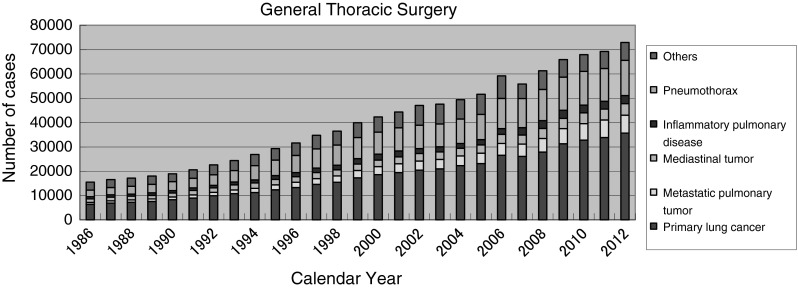
General thoracic surgery

